# Circulating tumor cells: from new biological insights to clinical practice

**DOI:** 10.1038/s41392-024-01938-6

**Published:** 2024-09-02

**Authors:** Xuyu Gu, Shiyou Wei, Xin Lv

**Affiliations:** 1 Department of Oncology, Shanghai Pulmonary Hospital, School of Medicine, Tongji University, Shanghai, China; 2 Department of Anesthesiology, Shanghai Pulmonary Hospital, School of Medicine, Tongji University, Shanghai, China

**Keywords:** Cancer metabolism, Cancer metabolism

## Abstract

The primary reason for high mortality rates among cancer patients is metastasis, where tumor cells migrate through the bloodstream from the original site to other parts of the body. Recent advancements in technology have significantly enhanced our comprehension of the mechanisms behind the bloodborne spread of circulating tumor cells (CTCs). One critical process, DNA methylation, regulates gene expression and chromosome stability, thus maintaining dynamic equilibrium in the body. Global hypomethylation and locus-specific hypermethylation are examples of changes in DNA methylation patterns that are pivotal to carcinogenesis. This comprehensive review first provides an overview of the various processes that contribute to the formation of CTCs, including epithelial-mesenchymal transition (EMT), immune surveillance, and colonization. We then conduct an in-depth analysis of how modifications in DNA methylation within CTCs impact each of these critical stages during CTC dissemination. Furthermore, we explored potential clinical implications of changes in DNA methylation in CTCs for patients with cancer. By understanding these epigenetic modifications, we can gain insights into the metastatic process and identify new biomarkers for early detection, prognosis, and targeted therapies. This review aims to bridge the gap between basic research and clinical application, highlighting the significance of DNA methylation in the context of cancer metastasis and offering new avenues for improving patient outcomes.

## Introduction

The progression of cancer from a localized tumor to a widespread metastatic disease is a complex and multifaceted process, making it cancer patients’ leading cause of death.^1^ The driving force behind this process is CTCs, which break from the primary tumor site and spread through the bloodstream to colonize other organs.^2^ The study of CTCs has garnered considerable attention as it opens new avenues for understanding the intricacies of metastasis, offering potential markers for early detection and targets for therapeutic intervention.^3^

Recent technological advancements in genomics and molecular biology have significantly enhanced our capability to scrutinize the biological mechanisms underpinning CTC dissemination.^4^ Among the myriad of molecular processes implicated in this journey, DNA methylation emerges as a pivotal regulatory mechanism influencing gene expression and chromosome stability.^5^ Cellular differentiation, growth, and adaptation to environmental changes all depend on DNA methylation, a reversible epigenetic alteration that adds methyl groups to the DNA molecule.^6^ For their roles in the development and propagation of cancer, changes in DNA methylation patterns are becoming more and more recognized.^7^

Hypomethylation across the entire genome, alongside hypermethylation of specific gene loci, has been implicated in the disruption of genomic integrity and the silencing of tumor suppressor genes, respectively.^8,9^ These epigenetic changes are fundamental to the transformation of normal cells into malignant ones, affecting their ability to proliferate, evade immune detection, and metastasize.^10^ This comprehensive review delves into the mechanisms of CTC formation, highlighting processes such as EMT, immune system evasion, and the colonization of distant tissues, all of which are critical to the metastatic cascade. Furthermore, this review aims to dissect the role of DNA methylation modifications within CTCs, examining how these alterations influence the aforementioned stages of CTC dissemination. After comprehending the epigenetic landscape of CTCs, researchers can find new targets for therapeutic intervention as well as biomarkers for cancer diagnosis and prognosis. The modulation of DNA methylation in CTCs, in particular, presents a promising path for the development of targeted therapies intended to prevent the spread of cancer.

## Biology of CTCs

**Fig. 1:**
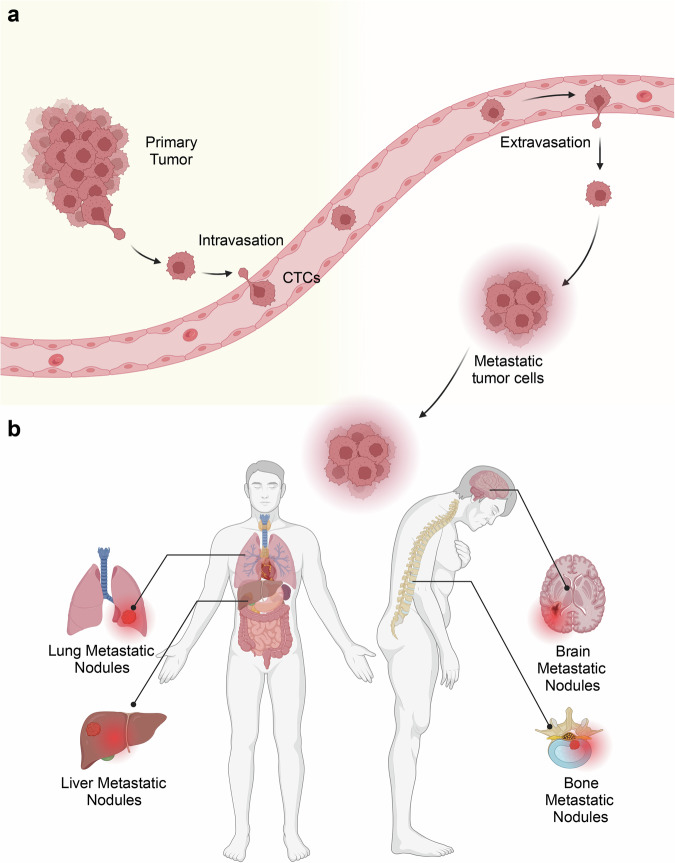
Route and location of CTCs Metastasis. Circulating tumor cells (CTCs) are the fundamental constituents of liquid biopsy, functioning as the cornerstones of this approach. **a** These neoplastic cells are shed naturally from primary or metastatic tumors and circulate within the bloodstream, **b** serving as the “seeds” of tumors that can potentially result in fatal metastasis of various sites. The figure was created with BioRender.com

CTCs enter the peripheral circulation from primary or metastatic lesions either spontaneously or as a result of therapeutic manipulation.^11^ The notion of CTCs was initially introduced by Ashworth in 1969, and these cells can be categorized into three groups: epithelial, mesenchymal, and hybrid.^12–15^ While most CTCs can be eliminated by the host immune system, a subset of highly active and metastatic CTCs may evade immune clearance, ultimately resulting in the establishment of microscopic cancer foci, tumor recurrence, and metastasis.^16–21^ Thus, CTCs are regarded as a plausible origin of fatal metastatic disease in individuals (Fig. 1).

However, there are several obstacles that CTCs must overcome in order to successfully detach from the primary tumor tissue, including: 1) Adhesion: Extracellular matrix (ECM) and surrounding cells in the tissue of the primary tumor are both firmly adhered to by cancer cells, which are typically highly sticky. CTCs must overcome these adhesion forces in order to detach and enter the lymphatic or bloodstream system. 2) Invasion: Cancer cells must also be able to invade the neighboring tissue to reach the lymphatic or blood vessels. This requires the cells to degrade the ECM and surrounding tissues. 3) Shear stress: Once in the bloodstream or lymphatic system, CTCs are subjected to significant shear stress due to the movement of fluid. This can cause mechanical damage to the cells and can also trigger apoptosis (cell death). 4) Immune system surveillance: The immune system is constantly surveilling the bloodstream and lymphatic system for foreign or abnormal cells. CTCs must avoid being detected by the immune system in order to survive and potentially form secondary tumors. Overall, the process of CTC detachment and dissemination is complex and involves many different factors. Understanding these obstacles and how CTCs overcome them is a crucial area of research on the biology of cancer and may lead to new therapies for preventing or treating cancer metastasis.

### Molecular markers of CTCs

CTCs have been found in a wide range of cancer types using a comprehensive panel of molecular markers. Table 1 provides a summary of CTC-associated markers utilized in different cancers.^22,23^ The leading marker for CTCs is epithelial cell adhesion molecule (EpCAM), a “universal” epithelial marker for tumors, since the majority of tumors originate in epithelial tissues.^24^ Different cancer types^25^ express EpCAM differently, and EpCAM-based CTC detection systems are widely employed for tumors with significant EpCAM expression, like prostate and breast cancers. Numerous studies have demonstrated that CTCs in prostate and breast cancers are EpCAM-positive, indicating their prognostic value in the disease’s early and metastatic stages.^26,27^ Other epithelial-derived cancers, including pancreatic,^28^ colorectal,^29^ and hepatocellular cancers,^30^ also exhibit significant detection rates of EpCAM-positive CTCs. Similar to early distant metastases, the presence of these EpCAM-positive CTCs is associated with poorer patient survival.^29,31,32^ However, there are limitations to using EpCAM as a CTC marker. Tumors that are low-expression or EpCAM-negative, including neurogenic cancers, are unable to be treated with this method. CTCs can undergo EMT, a process during which epithelial markers, including EpCAM, are downregulated. The rate of EpCAM-positive CTC detection is affected by this downregulation. Numerous studies have shown the clinical usefulness of EpCAM-positive CTCs, despite uncertainties about the efficacy of EpCAM-based technology for detecting all CTCs.^33^ Positive EpCAM CTCs make up a significant subset of all CTCs, indicating that they could serve as a trustworthy biomarker for cancer prognosis and therapy efficacy when necessary. EpCAM-positive CTCs are likely to miss critical biological information associated with EpCAM-negative CTCs, leading to an underestimation of the actual number of CTCs within the population. This phenomenon has been observed in certain types of cancer such as NSCLC (NSCLC), the number of EpCAM-negative CTCs is significantly higher than that of EpCAM-positive CTCs.^34^ However, the usage of both mesenchymal and epithelial cells, along with marker-independent detection techniques, can enhance the suboptimal isolation of CTCs by EpCAM-based technologies. For example, the use of fluorescent-magnetic nanoparticles with a dual-antibody interface that targets both N-cadherin and EpCAM in breast cancer has accelerated the identification of CTCs and increased the efficiency of CTC isolation.^35,36^ The identification of both epithelial and non-conventional CTCs, which lacked leukocyte and epithelial markers, enhanced the CTC positivity rate in a single-cell assay for CTC detection in biliary tract cancer.^37^

**Table 1:** Molecular markers of CTCs

Cancer types	Epithelial markers	Mesenchymal markers	Stemness marker	Specific markers
Breast cancer	EpCAM/CK	Vimentin	ALDH1	HER2
	CK	Twist	CD44	ER
	E-Cadherin	Fibronectin		AR285
		N-Cadherin		MRP48
		SERPINE1/PAI19		
		β-catenin281		
Prostate cancer	EpCAM/CK8,18,19	Vimentin	-	PSMA
		Twist		PSA
				EGFR
				ARV7
				PIM1
				AR v567es
Kidney cancer	EpCAM	-	-	CD147296
Bladder cancer	EpCAM/CK8,18,19	-	-	-
Colorectal cancer	EpCAM/CK8,18,19	Vimentin		PI3K α
		Twist		CEA
		SNAI1		PRL3
		AKT2		
		LOXL3		
		Plastin3		
Non-small-cell lung cancer	CK7/8/18/19	Vimentin	ABCG2	Folate receptor
	EpCAM/CK8,18,19	Twist	CD44	Telomerase activity
		N-Cadherin	ALDH1	
		AXL		
Small-cell lung cancer	EpCAM/CK8,18,19	Vimentin	ABCG2	DLL3
Pancreatic cancer	EpCAM/CK8,18,19	Vimentin	ABCG2	-
		Twist	CD44	
		KLF8		
Hepatocellular carcinoma	EpCAM/CK8,18,19	Vimentin	ABCG2	GPC3
	EpCAM,CK19346	Twist		ASGPR
	EpCAM348	-		-
Gastric cancer	EpCAM/CK	Vimentin	ABCG2	XAF
	CK19/CK20	N-Cadherin	CD44	MT1-MMP
				Survivin
				HER
Esophageal cancer	EpCAM/CK8,18,19	-	-	-
Cervical cancer	EpCAM/CK8,18,19	-	-	-
Melanoma	-		-	MART
				HMW-MAA
				CD146
				MAGE A
				GalNAc-T
				MAGE A1-6
				hTERT
				MLANA
				B4GALNT1
				PAX3
				DCT

Molecular changes accompany the EMT in cancer cells. Among these are the downregulation of epithelial indicators like ZO-1, claudins, and occludins and the upregulation of mesenchymal markers like Vimentin, N-cadherin, fibroblast-specific protein 1, and fibronectin.^38^ EMT is regulated by EMT-related transcription factors, mainly from the TWIST, ZEB, and SNAIL families.^39^ All these EMT-related molecules have the potential to be utilized in targeting methods for EMT-associated CTCs. Many EMT-related molecules, however, are nuclear or cytoplasmic proteins, limiting their application in current membrane-based CTC detection techniques. The usage of proteins like E-cadherin, Vimentin, and TWIST has been common in the past because of their detectability in traditional CTC detection methods, including as flow cytometry sorting, immunostaining, and fluorescence in situ hybridization (FISH) staining.^40^ Nevertheless, the advent of single-cell CTC sequencing technologies^41^ holds the promise of more comprehensively revealing the EMT status of CTCs, encompassing all EMT-related molecular alterations at the RNA level.

Numerous studies have proposed that either differentiated progenitor cells or somatic stem cells may be the source of cancer stem cells (CSCs) with tumor initiating capacities. Evidence suggests that this specific subpopulation of cancer cells may be responsible for relapse and metastasis.^42^ If the majority of cancer cells are unable to form new tumors and only the rarest CSCs may travel to cause metastatic disease, therefore the main goal of CTC research is to identify and eliminate this circulating-CSC population. After identifying and isolating CSCs within CTCs, it would be possible to combat residual cancer more effectively. Two hypotheses can be taken into consideration to clarify the origin of circulating CSCs. First, cancerous somatic stem cells EMT and exit the main tumor into the circulation. These cells are known as mesenchymal CSCs. Second, these cells can be the result of fully differentiated cancer cells that have undergone EMT pathways to acquire migratory properties.^43^ Regardless of the pathway, the end result is the dispersal of CSCs, which move in the direction of niches via intermediary cells that display a combination of mesenchymal, epithelial, and stemness characteristics. This could explain the diversity of surface markers and/or transcription factors observed in tumor-initiating cells within CTCs. The heterogeneity of CTCs, originating from either EMT in CSCs or differentiated cancer cells, underscores the challenge of selecting significant identification markers. This complexity is further compounded by the specific organ of tumor origin. During EMT, cancer cells acquire stemness characteristics, transforming into mesenchymal stem cancer cells. The MET, which is a reverse process, transforms them into epithelial stem cancer cells. ALDH1 expression, initially observed in 1–2% of normal breast epithelial cells, was first documented in breast cancer tissue by Ginestier et al.^44^ Barriere et al. found in a clinical study for early breast cancer diagnosis that blood samples from patients with non-metastatic breast cancer had a stemness CTC population. Out of 130 patients, 17 had ALDH1 markers and 49 were CTC-positive.^45^ CD44, a cell surface glycoprotein implicated in metastasis and cell migration,^46^ characterizes a subpopulation of breast CSCs capable of intravasating into the bloodstream, identified by the CD44^+^/CD24^low/–^/Lin^–^markers.^47^ In colorectal cancer, CSCs expressing CD44v6 are associated with metastatic potential.^48^ Additionally, CSCs have decreased levels of certain glycosphingolipids that are important for cell growth and motility, such as Fuc-(n) Lc4Cer and Gb3Cer, while elevated levels of GD2, GD3, GM2, and GD1a are seen. These gangliosides have the potential to function as markers of CTC stemness. Specifically, GD2 is associated with the CD44high CD24low phenotype linked to CSCs, and GD3 similarly indicates stemness features, although GD1a remains a putative marker.^49,50^ Recent research has also linked the CSCs phenotype to high ABCG2 transporter expression levels. This transporter is responsible for removing a variety of xenobiotics, including chemotherapy drugs. This chemoresistance implies that ABCG2 expression and stemness are closely associated. It has been found that CSCs from lung, pancreatic, and retinoblastoma tumors exhibit high levels of ABCG2.^51,52^ Additional biomarkers have been found to be markers for CTCs in a variety of cancers, each with unique therapeutic implications. These include human epidermal growth factor receptor-2 (HER-2)^53,54^; estrogen receptor^55–57^; prostate-specific membrane antigen^58,59^; folate receptor^60,61^; and survivin.^62^ Table 1 provides specifics on these CTC markers. The majority of these markers match the unique molecular markers of the primary tumor. However, the expression of specific markers in CTCs often differs from that of the primary tumor. For example, there is about a 15%^63^ discrepancy in HER-2 gene amplification between primary breast tumors and CTCs, indicating that genetic instability is most likely the cause of clonal selection or acquisition. CTC detection technologies are dependent on a number of melanoma cell adhesion molecules, which are highly specific molecular markers for melanoma and include HMW-MAA,^62,64^ MART-1,^65,66^ CD146,^67,68^ and MAGE A.^66,67,69–71^ Melanoma is a kind of skin cancer that starts in melanocytes. The diverse range of CTC markers underscores the heterogeneity of CTCs across various cancer types. Even within a single patient, CTCs display spatio-temporal heterogeneity, possibly attributable to spatially distinct microenvironments within the bloodstream and temporal variations in response to therapy. As such, characterizing the whole population of CTCs using the few molecular markers that are currently accessible is difficult. Furthermore, CTC markers vary over the course of cancer treatment and at different stages of the disease.

### The biological progress of CTCs development

Epithelial cells typically undergo anoikis, a form of programmed cell death, upon detachment from their surrounding environment. This characteristic makes metastatic seeding generally inefficient.^72–74^ This raises an important question: what characteristics allow CTCs to metastasize successfully? Enhanced survival and tumor-seeding ability appear to be restricted to a small subset of cells initiating tumors or metastases with stem-like properties.^75^ EMT has been proposed as an essential factor for metastasis since it increases both invasive potential and contact-independent survival (i.e., resistance to anoikis).^75–77^ Preclinical studies have shown that EMT transcription factors, like TWIST and SNAIL, enhance motility and invasiveness in vitro while suppressing cell-cell adhesion.^78,79^ Silencing SNAIL and TWIST, however, can reduce metastasis in vivo but does not totally prevent it, raising doubts about the absolute requirement of EMT-related transcriptional regulators in the metastatic process.^80,81^ Interestingly, it has been found that EMT can inhibit the effective seeding of metastatic tumors by deleting E-cadherin or modifying TWIST expression in several EMT lineage tracing models.^80,82^ The observed plasticity of the epithelium-to-mesenchymal transition raises the possibility that this change is fluid and transitional rather than binary and irreversible, acting more as a catalyst for metastasis than as a direct cause.^83,84^ Consequently, CTCs have been seen to exhibit an intermediate degree of EMT, which is linked to their adaptability, stem cell-like qualities, poor response to therapy, and progression of the disease.^76,84,85^ There have been multiple hybrid states found at the invasive boundary of patient tumor tissues from common carcinoma types and xenografts.^84,86,87^ Their functional role in invasion, invasion, and metastatic colonization requires further studies.

There may be concurrent existence of metastasis pathways that are both plasticity-dependent and plasticity-independent.^88^ The discovery of collectively moving, highly metastatic clusters of cell contact-dependent CTCs, which were first reported several decades ago, offers a significant refutation of the idea that EMT is necessary for metastasis.^89,90^ Metastatic colonies are polyclonal,^91–94^ and subclones interact synergistically,^95,96^ suggesting that heterogeneous CTC clusters^97,98^ as well as solitary CTCs are involved in cancer propagation. Despite making up a small portion of all CTC events in peripheral circulation, multiple studies have demonstrated that CTC clusters have up to a 100-fold higher potential for metastasis than individual CTCs.^82,97–99^ Intratumor hypoxia is shown to upregulate genes producing cell adhesion proteins in patient samples and mice models, which promotes collective CTC cluster shedding.^98^ Homotypic clustering has been shown to improve a number of cellular characteristics, such as the overexpression of stem-like traits^100,101^ via the hypomethylation of binding sites for transcription factors OCT4, SOX2, and NANOG^102^ and the methylation of metastasis suppressor genes.^5^ Clustering can also be augmented by circulating galectin 3 or cancer-associated MUC1,^100^ homotypic ICAM interactions,^103^ or CD44 interacting with PAK2.^98^ This clustering can also enhance survival and self-renewal capacity through CDK6 or increased size or number of desmosomes and hemidesmosomes.^97,104^ CD44 was among the first markers discovered to detect breast cancer cells with elevated tumor-initiating capacity in solid tumors.^47^ Later, it was demonstrated that CD44, along with MET, epithelial EpCAM, and CD47, could identify subpopulations of breast cancer that were highly metastatic.^76^ Importantly, CD44 expression should not be employed as a CTC marker alone because it is widely expressed in the hematopoietic cell compartment.^105^ Previously thought to be specific to stem-like cells, CK14 expression is more common in CTC clusters than in individual CTCs and is required for distant metastasis.^106^ EpCAM^–^, HER2^+^, EGFR^+^, heparanase (HPSE)^+^, and NOTCH1^+^ are stem-like phenotypic signatures in CTCs that have been shown to provide competence for brain and lung metastasis.^104^

Heterotypic clusters, which include platelets, myeloid cells, and cancer-associated fibroblasts (CAFs), as well as homotypic clusters formed by CTCs, can potentially form between CTCs or other cell types.^107–111^ The rapid interaction of CTCs with platelets in circulation^112^ enhances plasticity and metastasis-initiating capacity^107^ through mechanisms like RhoA–MYPT1–PP1-mediated YAP1 signaling^113^ and increased vascular permeability via platelet-derived ATP–P2Y2 interaction.^114^ The glycoprotein A additionally, protecting against T cell-mediated clearance and NK cell-mediated clearance, respectively, are repetitions predominant (GARP)–TGFβ axis^115^ and platelet-derived major histocompatibility complex class I.^116^ Barbara Maria Szczerba et al. demonstrated that neutrophils also participate in forming heterotypic CTC clusters.^117^ Neutrophils are drawn to CTCs by chemotaxis that is dependent on CXCL5 and CXCL7^111^ and connects with them via adhesion mediated by vascular cell adhesion molecule 1 (VCAM1), which enhances their potential for proliferation and metastasis.^111^ Through the formation of neutrophil extracellular traps or the secretion of matrix metalloproteinases and IL-1β, neutrophils facilitate adhesion and extravasation.^108^ In addition, neutrophils protect CTCs from immune surveillance^118,119^; macrophages and myeloid-derived suppressor cells in CTC clusters also provide this protection.^118,120^ It has been demonstrated that heterotypic adherens junctions, mediated by N-cadherin in stromal CAFs and E-cadherin in invasive cancer cells, promote collective invasion. Here, CAFs serve as invasive and migrating leader cells,^110,119^ facilitating metastasis by allowing tumors to “bring their own soil”.^121^ Mathematical models indicate that the form of clusters influences CTC behavior during circulation in addition to the biological consequences of clustering. Compact clusters usually flow closer to the endothelium wall compared to linear clusters.^122^ However, clusters move in a “single chain” structure as they pass through narrow capillaries.^109,123–125^ The metastatic potential of CTCs is significantly impacted by the phenotypic plasticity and clustering differences, which highlights potential strategies for inhibiting the metastatic process.

Furthermore, the dynamics of CTC propagation are becoming recognized as an equally important component for the dissemination of tumor cells.^111,126^ Chronotherapy has been used to study and evaluate the clinical application of the circadian rhythm’s function in tumor onset^127,128^ and growth dynamics.^129,130^ This therapeutic approach seeks to optimize therapy timing in order to increase the effectiveness of antitumor drugs.^131,132^ However, it has only recently been established how circadian rhythm impacts CTC release and the dissemination of metastatic disease.^111,126^ The way that CTCs are currently detected frequently assumes that peripheral blood counts remain stable throughout the day, which may lead to inconsistent results and make it more difficult to use CTCs as a liquid biopsy analyte in clinical settings. Fluorescence in vivo flow cytometry studies in orthotopic mice models of human prostate cancer have suggested circadian rhythmicity.^133^ The temporal dynamics of CTC intravasation, demonstrating significant circadian rhythm-based variations both in breast cancer patients and in mouse models. CTC counts are highest during sleep because to these variations, which are caused by rhythmic changes in hormone levels, such as melatonin.^128^

### The role and mechanism of CTC in drug resistance

The manifestation of an EMT phenotype in CTCs has been associated with therapeutic resistance and tumor relapse. For example, in patients with metastatic colorectal cancer, overexpression of CSV and plastin 3 in EMT-positive CTCs has been associated with drug resistance.^134^ CTC survival is improved by the acquisition of stem cell characteristics during EMT, which promotes enhanced migratory and invasive abilities as well as resistance to therapy. Tumor relapse following targeted therapy can be driven by CTCs with stem cell features,^135^ such as in colorectal cancer,^136^ where drug-resistant CTCs may act as metastasis-initiating cells, driving tumors to become more aggressive as a result of anticancer drugs’ selection pressure. Targeted therapy-induced tumor relapse in melanoma patients has been linked to phenotypic switching of CTCs to a less differentiated state.^137^ Therapies resistance in breast, prostate, pancreatic, and NSCLC have also been linked to phenotypic switching.^138^ Therapeutic resistance mechanisms seen in primary tumor cells include target mutation or inactivation, improved genomic DNA repair, faster drug efflux, upregulation of markers associated with quiescence, downregulation of markers associated with proliferative activity, and inhibition of the formation of oxygen radicals,^139^ are also evident in CTCs. Tumor cells can separate from the original tumor and circulate in the blood as clusters of tiny cells, challenging the idea that CTCs with EMT and/or stem cell phenotypes are the sole type that initiates metastasis (i.e., groups of >2 CTCs and up to large micro-emboli).^97^ Metastatic potential and drug resistance may be influenced by CTC clusters. The absence of proliferation in CTC clusters, as indicated by Ki67 staining, has been linked to their resistance to cytotoxic therapies.^140^ Significantly reduced overall survival following systemic therapy is observed in PDAC patients who harbor a high number of CTC clusters (more than 30 clusters per 2 mL of blood).^141^ Expanding CTCs from patients into cell lines or xenografts may reveal insights into their therapeutic resistance and present an opportunity for targeted therapy.^142^ However, high CTC numbers and several months of establishment make CTC-derived cell lines or xenografts impractical for clinical use. Furthermore, prolonged passage of CTCs in culture is likely to induce irreversible adaptation and clonal expansion.^143^

Stem cell and EMT markers exhibit frequent overexpression in CTCs of metastatic breast cancer.^144^ Recently, within breast tumors, a subpopulation displaying a stem cell-like phenotype characterized by CD44 positivity and CD24 negativity has been delineated.^47^ This subpopulation potentially disseminates into the bloodstream, evading therapeutic measures,^145^ and demonstrates an expression profile associated with metastatic recurrence.^146^ The modulation of HER2 signaling has been reported to augment cancer stem cell reservoir, potentially necessary for its maintenance; a significant association between HER2-like tumors and stem cells has been observed.^147^ A breast cancer stem cell-like phenotype, exhibiting higher resistance to treatment and reduced proliferation in circulation, is suggested by preliminary evidence.^148^ ALDH1, a marker indicative of both normal and neoplastic breast stem cells,^144^ has been found to be overexpressed in 70% of CTCs, correlating with therapy resistance. A substantial proportion of CTCs display detectable levels of at least one EMT marker such as TWIST, AKT2, and PI3Kalpha, in addition to ALDH1, thereby delineating a highly tumorigenic subset of EMT-associated breast CSCs.^44^ This subset of CTCs has clinical value since it predicts drug resistance and a poor result for patients with metastatic cancer.^44^ Notably, ALDH1 and EMT markers were discernible even prior to CTC detection in circulation, as evidenced by their positivity in RT-PCR for transcripts such as HER2, MUC1, and EpCAM.^44^ Consistently, stem cell-like phenotypes, particularly CD44^+^CD24^−/low^ and ALDH1^high^CD24^−/low^, have been found in 35.2% and 17.7% of CTCs, respectively.^63^ Among ALDH1-highly positive CTCs, the subset expressing CD44^+^CD2^4−/low^ exhibits heightened tumorigenic potential.^149^ Though ALDH1-positive cells constitute just 5% of cells in tumors expressing ALDH1, ALDH1 positivity is associated with a high histological grade and poor clinical outcomes.^149^

According to the cancer stem cell model, putative cancer stem cells^44^ are necessary for tumor growth and drug resistance, and their existence should be associated with a worsened prognosis,^149^ albeit data on this matter are still inconsistent.^44,150,151^ Because of their capacity to self-renew and resistance to chemotherapeutic drugs, eradicating these cells during therapy poses challenges. Notably, patients’ chances of responding to chemotherapy are poorer when their CTCs are ALDH1-positive.^44^ It has recently been shown that CTCs express one or more multidrug resistance-related proteins (MRPs) in 86% of metastatic breast cancer patients, with patients exhibiting positive MRP-positive CTCs having considerably shorter progression-free intervals.^57,144,152,153^ Further investigations are warranted to elucidate the association between the presence of CD44^+^CD24^−^ or ALDH1^high^CD24^−/low^ CTCs and clinical trajectory as well as disease progression.

### CTC isolation and identification

For CTCs to be used as a liquid biopsy analyte in clinical settings, it is essential to adopt impartial, affordable, quick, and effective capture technologies that can reliably isolate adequate numbers of CTCs. In order to facilitate the development of data necessary for precise patient stratification and therapeutic decision-making, these capture approaches must also be compatible with functional assays and cutting-edge sequencing technologies. For probing the biology and vulnerabilities of metastatic cancer at the molecular and functional levels, CTCs provide a wide variety of scope when they are isolated in a viable state. CTCs have a unique role as a liquid biopsy analyte because they can represent aggressive subclones with greater metastatic potential. There is conjecture that their molecular and phenotypic analysis may provide more pertinent insight than traditional tissue biopsies (which involve the random subclone isolation)^154,155^ or other circulating analytes like circulating tumor DNA (which mainly identify dying subclones). However, further exploration is warranted. The ease of access provided by minimally invasive blood draws may allow for regular, long-term assessments of the effectiveness of treatment interventions, which may also make early cancer detection or recurrence possible.^156^ As a result, CTCs become an optimal biomarker repository for personalized therapy and real-time clinical applications. However, it is still challenging to obtain CTCs because of their paucity, and effective CTC enrichment is required for reliable downstream analysis and applications.

In the previous ten years, numerous technical progress has been made to improve CTC analysis and detection^157,158^ by utilizing distinct characteristics and phenotypes of CTCs. These developments can be broadly categorized into antigen-dependent and antigen-independent techniques. The most widely used strategies currently in use help to promote positive selection by using antigens that are expressed on CTCs and minimally expressed on other circulating cells. This strategy is frequently combined with CD45-based negative selection to reduce the number of hematopoietic cells and enhance discrimination. It is notable that the CellSearch system (Menarini Silicon Biosystems, Italy) and AdnaTest CTC Select (QIAGEN, Germany), which have been approved by the US Food and Drug Administration (FDA), utilize immunomagnetic selection based on EpCAM expression.^159,160^ To increase sensitivity and specificity, respectively, more markers are used, such as pan-CK and CD45. For CTC capture, antibody-coated magnetic beads are used in the Magnetic-Activated Cell Separation (MACS) method (Miltenyi Biotec, Germany).^85^ Additionally, the Geometrically Enhanced Differential Immunocapture method combines microfluidics with different antibodies according to the kind of tumor (tumor includes, for instance, HER2 in breast cancer and PSMA in prostate cancer) and positive for cytokeratin for counting.^161^

Physical characteristics including size, charge, density, or elasticity are used by antigen-agnostic detection systems to enrich CTCs. Detection of CTCs based on physical properties is facilitated by several methods, including density gradient centrifugation, filter-based devices, capture surfaces, and microfluidic systems. Notable examples of microfluidic systems are ISET (Rarecells Diagnostics, France), Smart Biosurface Slides, CTC-iChip (TellBio, USA), and the FDA-approved Parsortix (ANGLE, UK).^162,163^ To improve sensitivity and specificity even more, multimodality techniques are being developed. For example, Isoflux (Fluxion Biosciences, USA) combines immunomagnetic beads,^164^ with flow control, and the Cyttel system (CYTTEL Biosciences, China) is an image-based detection tool that identifies CTCs by combining immunohistochemistry, fluorescence in situ hybridization, and centrifugation in that order. With the use of the microfluidic platforms Parsortix and CTC-iChip, marker-based positive and negative selection (such as EGFR, HER2, CD45, and EpCAM) can be combined with imaging and micromanipulation to identify pure CTC subsets.^5,111,162,165^

Novel in vivo CTC detection tools have emerged to address the challenge of low CTC levels in peripheral blood samples. CTCs can be directly extracted from the bloodstream using methods such as intravascular CTC-catching guidewires coated with EpCAM-directed antibodies, as exemplified by the CellCollector device from GILUPI in Germany.^166^ When combined with antigen-dependent selection, cytopheresis facilitates the enrichment of cell fractions from vast blood volumes and offers promise for the isolation of CTCs.^167^ The adoption of this approach into routine clinical practice may pose challenges due to its lengthy and invasive nature, alongside potential vascular health issues in heavily treated cancer patients. Studies comparing different methods of accessing the vasculature shows that patients with early-stage NSCLC have higher CTC counts in tumor-draining vessels than in peripheral locations.^156,168^ For early-stage cancer patients undergoing surgery, this concept provides an attractive opportunity for liquid biopsy. Despite its significance, it remains impractical to use these findings to routine CTC evaluation or to patients with advanced illness who do not undergo surgery.

Advances in capture methods have enabled thorough molecular and functional studies of CTCs at both bulk and single-cell levels, epigenomic, exploring genomic, proteomic, transcriptomic, and functional properties. This has extended research on these cells beyond mere counting to comprehensive analysis. Since these advances have been extensively reviewed, we will focus primarily on aspects related to cell multi-omics and the functional evaluation of CTCs.^2,157,169,170^ For instance, assessing drug responses alongside single-cell examination of individual CTCs and CTC clusters can uncover biological dependencies and potential targets for therapy.^5^ CTC proteins and secreted factors are being characterized using microfluidic platforms that employ single-cell resolution mass spectrometry and bead-based immunoassays.^171–173^ A key barrier to ex vivo CTC cultures’ clinical applicability is their low success rates, despite the fact that experimental proof-of-principle studies^174,175^ have demonstrated their efficacy. Although the feasibility and therapeutic relevance of CTC capture and downstream analysis have been shown, most of the above approaches are not currently used as routine. Existing CTC technologies face limitations that necessitate resolution for robust integration into clinical settings. These issues encompass the necessity for a more profound comprehension of epitope expression and plasticity, along with challenges associated with cell loss caused by variations in size and deformability, low purity of CTCs, device blockage, the need for substantial blood volumes, time limitations, and difficulties with automation. Additional challenges include the need to improve functional assays, such as enhancing culture methods and developing CTC-derived xenografts, and ensuring robust validation of molecular analyses. This involves addressing issues like stochastic variations, limited sequencing coverage, biases in amplification, high error rates, and variability in bioinformatics approaches.^2^ If these challenges are overcome, CTCs may become prominent sources of comprehensive biomarkers that are minimally invasive and personalized.

## Metastasis of CTCs

Stephen Paget first proposed the “seed theory” or the soil and seed hypothesis of metastasis in 1889.^176^ According to the hypothesis, cancer cells (seeds) have a selective affinity for certain organs (soil) and that the capacity of cancer cells to establish colonies in distant locations is determined not only by the characteristics of the cells themselves but also by the specific microenvironment of the secondary site. According to the seed theory, cancer cells must go through a sequence of steps in order to successfully metastasize to a distant site. encompass the cancer cells’ ability to invade the nearby tissue, penetrate the bloodstream or lymphatic system, survive while circulating, extravasate into the secondary site, and establish a new colony of cancer cells.

### Initiation of metastasis

**Fig. 2:**
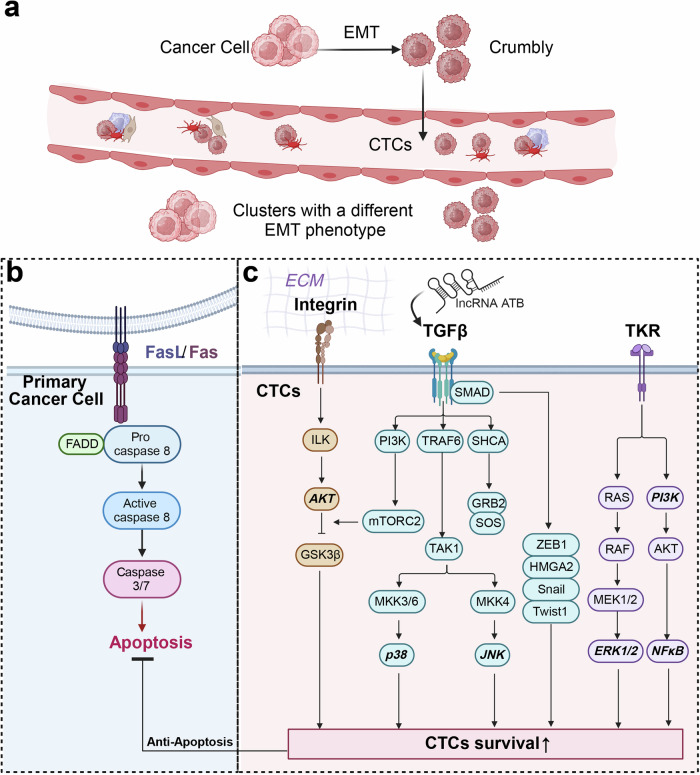
Schematic representation of EMT-associated mechanisms supporting CTC survival and early metastasis. **a** CTCs are liberated into the bloodstream by means of epithelial-mesenchymal transition (EMT)-related mechanisms, including individual cell or collective migration, intravasation, as well as passive processes such as the detachment of isolated tumor cells or clumps through damaged blood vessels. Certain individual or clustered CTCs have the ability to survive in the bloodstream and form metastases in secondary organs. This is due to their possession of characteristics that are elevated in EMT-induced cells, and that facilitate their survival in the bloodstream and establishment of metastases. Specifically, **b** the Fas/FasL signaling pathway plays a crucial role in tumorigenesis, where impairment in cancer cells can lead to resistance to apoptosis and contribute to tumor progression and CTCs generation, **c** EMT progression is regulated by signaling pathways such as integrin and TGF-β that can cooperate to induce downstream responses to promote CTCs survival and anti-apoptosis properties. The figure was created with BioRender.com

**Fig. 3:**
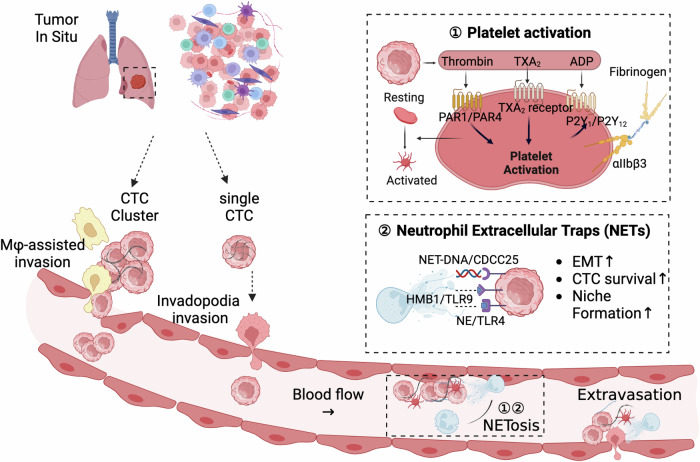
Mechanisms of CTCs cluster generation and the potential CTC subpopulations survival in the blood circulation. CTCs that undergo EMT express Tissue Factor (TF), which significantly contributes to platelet activation. These coagulation-dependent mechanisms initiate the formation of a fibrin/platelet-rich cocoon around tumor cells, believed to offer protection from shear stress, anoikis, and immune attack. The formation of the aforementioned cocoon is crucial for CTC seeding and early establishment. Additionally, neutrophils have been observed to physically interact with tumor cells and platelets, thereby promoting tumor cell survival and proliferation. Furthermore, neutrophils aid in the adhesion of CTCs to the vascular wall. Neutrophils, through their capacity to capture tumor cells in Neutrophil Extracellular Traps (NETs)—structures that also facilitate coagulation events - aid in the formation of a protective and anchoring scaffold that supports CTC survival. This process facilitates the arrest of CTCs in capillaries and early phases of metastatic establishment. The coagulation- and neutrophil-dependent shielding mechanisms described above safeguard CTCs from immune destruction. Furthermore, CTCs, especially those that undergo EMT, possess an increased ability to evade immune surveillance. One mechanism that contributes to this is the expression of immune checkpoint proteins such as PD-L1, which likely enhances their resistance to cytotoxic immune cells. After a possible period of dormancy, CTCs can eventually resume growth and initiate secondary tumors. The figure was created with BioRender.com

According to the “seed theory”, metastasis typically entails two distinct steps. The first step entails a tumor cell’s detachment from its original site and subsequent circulation through the bloodstream or lymphatic system to a distant location. The second step involves the successful colonization of the tumor cell in the distant site, which requires the cancer cell to be able to proliferate and establish a secondary tumor.^177^ CTCs are crucial for the process of tumor metastasis, and it is believed that a specific subgroup of CTCs found in patient blood is what initiates the metastatic cascade.^76^ During the early stages of cancer, cellular properties like adhesion and stroma formation act as physical obstacles that impede distant metastasis. To overcome these barriers, carcinoma cells must boost their motility within the stroma and gain access to the bloodstream through either active or passive entry mediated by either the EMT or non-EMT pathways. CTCs that are viable in the bloodstream have the potential to arrest at various locations, including the secondary metastatic sites, or new distant locations.^178–181^ Upon arrest, these CTCs have the potential to undergo MET, which can facilitate extravasation and allow the cells to either enter a dormant state or colonize and engraft at the site (Fig. 2). CTCs with stem-like properties, demonstrating increased resistance to anoikis and invasive potential, are identified as crucial for successful metastatic dissemination due to their phenotypic heterogeneity.^182^ Notably, CTC clusters, characterized by polyclonality and enhanced metastatic potential, exhibit superior survival in circulation compared to individual CTCs.^183^ These clusters are formed through homotypic interactions among tumor cells or heterotypic interactions involving other cell types such as platelets or myeloid cells.^184^ The formation of these clusters not only boosts the proliferation and survival of CTCs in the bloodstream but also enhances their metastatic efficiency by aiding in immune evasion and seeding competency at distant sites (Fig. 3). Key factors like intratumor hypoxia and platelet interactions are implicated in promoting CTC cluster formation and the initiation of metastasis.^185^

### EMT of CTCs

Through a process called EMT, tumor cells can acquire on mesenchymal characteristics like enhanced motility and invasiveness.^186–188^ The dynamic and intricate process of EMT in cancer cells involves changes in gene expression and the activation of various signaling pathways. During the early stages of EMT, cancer cells undergo a transformation in which they lose their epithelial traits, such as intercellular adhesion and polarity, and gain mesenchymal characteristics to acquire an invasive phenotype.^189,190^ Alongside this, a number of genes show changed expression, including those related to ECM remodeling, cytoskeletal organization, and cell adhesion.^191–193^ As the EMT process progresses, cancer cells may undergo additional changes, such as the activation of stemness, which can lead to tumor heterogeneity and treatment resistance. EMT can also facilitate the formation of metastases, as mesenchymal-like cancer cells are better able to invade neighboring tissues and enter the bloodstream or lymphatic system.^194,195^ The progression of EMT in cancer cells is influenced by various factors such as signaling pathways (TGF-β, WNT, and NOTCH, etc.) including the TME, such as hypoxia and inflammation.^38,196^ Furthermore, EMT is regulated by a number of transcription factors, such as Twist, Slug, and Snail, which have the ability to either activate or inhibit the expression of genes connected to EMT.^197–199^ In general, EMT in cancer cells is a dynamic and complex process that is orchestrated by multiple signaling pathways and transcription factors. Understanding the mechanisms that drive EMT in cancer cells may provide new ideas for the management of cancer.

#### Initiation of EMT

Several signaling pathways, such as TGF-β, WNT, and NOTCH signaling, initiate the process of EMT. These pathways trigger transcription factors, such as Twist, Snail, Slug, and zinc finger E-box binding homeobox 1 (ZEB1), that promote mesenchymal genes and suppress epithelial genes.^200–202^ By reducing cell-cell interaction, EMT-related transcription factors including Snail and TWIST promote motility and invasiveness in vitro. Nevertheless, knockdown of these factors could only partially limit metastasis in vivo, which challenges the necessity of them for the metastatic process.^80,81^ Interestingly, loss of E-cadherin or TWIST modulation was found to prevent the successful metastatic seeding of in several EMT lineage tracing models.^82^ Many studies have investigated several genes associated with EMT in CTCs. Among the commonly studied EMT target genes, Vimentin is often included.^203–205^ Vimentin, primarily expressed in mesenchymal cells as a type III intermediate filament, is a well-known indicator of EMT and is frequently studied in relation to CTCs. Moreover, it has been associated with tumor metastasis, including promoting CTC survival and tumor cell migration.^206,207^ EMT has been observed to affect the expression of a variety of epithelial adhesion molecules, thereby modifying intercellular interactions. A typical example is the modulation of adhesion molecules, which involves suppression of E-cadherin and up-regulation of N-cadherin. This change in expression has been linked to the EMT process,^208,209^ and both E-cadherin and N-cadherin, molecules that modulate cell-cell interactions and whose expression is altered in EMTs, are often evaluated in CTCs. The adhesion molecule EpCAM, previously utilized in CTC studies for enrichment purposes, is another EMT target gene that is frequently investigated in CTC research.^210,211^

#### Disassembly of cell–cell junctions

EMT begins with the disruption of tight junctions, adherens junctions, and desmosomes, which hold epithelial cells together.^111,212,213^ In order for tumor cells to separate from the main tumor and infiltrate nearby tissues, this step entails the breakdown of the intercellular adhesion molecules that hold epithelial cells together. One key player in the disassembly of cell-cell junctions during EMT is Snail.^214^ Snail suppresses the E-cadherin expression, which is a key component of adherens junctions that hold epithelial cells together. By recruiting co-repressors such histone deacetylases (HDACs) and chromatin remodeling factors, snail is able to repress this gene by attaching to E-box elements in the E-cadherin promoter region.^63,215^ This results in the E-cadherin silencing and the subsequent loss of cell-cell adhesion. Other transcription factors such as ZEB1 and Slug also exerts crucial functions in the disassembly of cell-cell junctions during EMT. Slug represses E-cadherin expression by binding with its promoter and recruiting HDACs and other co-repressors. ZEB1 upregulates mesenchymal genes including Vimentin and fibronectin while suppressing the expression of E-cadherin, further supporting the mesenchymal phenotype of cancer cells.^216,217^ A role for signaling pathways in the disassembly of cell-cell junctions during EMT has also been studied in previous research. For instance, it is known that the TGF-β pathway induces EMT in different forms of cancer by increasing the expression of associated transcription factors that inhibit E-cadherin.^218,219^ The Notch and Wnt pathways have also correlated with EMT and cell-cell junction disassembly in different cancer types.^114,220,221^ In conclusion, the disassembly of cell-cell junctions is a complex process that entails the dysregulation of multiple signaling pathways and transcriptional networks. The development of novel therapies for the control and prevention of metastatic cancer may result from improving our understanding of the molecular mechanisms underlying this process.

#### Remodeling of the ECM

During EMT, cancer cells undergo changes that allow them to break down the ECM and invade surrounding tissues.^191^ Several studies have investigated the molecular mechanisms underlying this process in various cancer types. One of the key players in ECM remodeling during EMT is the matrix metalloproteinases (MMPs). MMPs are a class of zinc-dependent endopeptidases that can degrade collagens, laminins, and proteoglycans, among various ECM components.^222–224^ MMPs are upregulated during EMT in numerous cancer types, and their expression is manipulated by a number of signaling pathways and transcription factors, including Twist and Snail.^225,226^ Other proteases have also been implicated in ECM remodeling during EMT, including urokinase-type plasminogen activator (uPA) and its receptor.^227,228^ uPA is an enzyme belonging to the class of serine proteases that can activate plasminogen, leading to the degradation of ECM components.^229^ uPA expression is upregulated during EMT in various cancer types, and its expression is governed by various transcription factors such as Snail and ZEB1.^230,231^ The remodeling of the ECM during EMT also involves changes in the expression and activity of integrins, a group of transmembrane receptors that regulate cell-ECM interactions. A variety of signaling pathways and transcription factors, such as TGF-β and Snail, orchestrate the upregulation of integrin expression during EMT in various cancer types.^232^ Integrins can promote the invasiveness of cancer cells by activating downstream pathways such as phosphatidylinositol 3-kinase (PI3K) and focal adhesion kinase.^233,234^ Studies have also shown the involvement of non-coding RNAs (nc), including long nc RNAs (lncRNAs) and microRNAs (miRNAs), in ECM remodeling during EMT. For example, miR-29b has been shown to inhibit the expression of MMP2 and MMP9, leading to the suppressed ECM degradation and inhibited EMT in lung cancer cells. Similarly, the lncRNA MALAT1 has been demonstrated to trigger ECM remodeling and EMT in bladder cancer cells by manipulating the expression of MMP2 and uPA.^235–237^

### Non-EMT with CTCs

Recent studies utilizing transgenic mouse models has indicated that invasion and metastasis may happen without the involvement of EMT. These findings support the concept that the initiation of metastasis may not necessarily require EMT.^80,81^ The deletion of EMT-related transcription in transgenic mouse models suggests that EMT inhibition does not affect the development of systemic metastasis.^81^ Moreover, it has been reported by Godinho et al. that cancer cell invasion can also be triggered by centrosome amplification. This occurs through the disruption of cell-cell adhesion and an increase in actin polymerization dependent on Arp2/3.^238^ Moreover, passive infiltration, which is caused by external forces such as mechanical stress and tumor growth, can lead to the genesis of “accidental” CTCs in the bloodstream.^239,240^ The disseminated cancer cells that arise from invasion not mediated by EMT can reside either as individual cells or clusters and maintain their epithelial phenotype.^80,239^

CTC clusters pose a higher risk for cancer metastasis compared to individual CTCs, and they also have unique properties that make them resistant to chemotherapy and immune surveillance. Though the mechanism of CTC cluster formation remains elusive, several studies have shed light on the process. A study by Aceto et al. investigated the role of E-cadherin, a cell adhesion molecule, in CTC cluster formation. They found that E-cadherin was downregulated in single CTCs, but it was upregulated in CTC clusters. Furthermore, they showed that blocking E-cadherin expression prevented CTC cluster formation, suggesting that E-cadherin plays a crucial role in CTC cluster formation.^241,242^ Gasic et al. was one of the first to suggest that platelets might be involved in CTC cluster formation.^84^ They found that platelets accumulated around CTCs in the bloodstream, and they speculated that platelets might facilitate CTC cluster formation. More recent studies have confirmed this hypothesis. For instance, a study by Haemmerl found that platelets promoted CTC cluster formation by activating the TGF-β signaling pathway, which upregulates EMT genes and downregulates cell adhesion molecules.^243,244^ The role of integrin in CTC cluster formation. They found that CTCs overexpressed several integrins, including integrin α5β1, which promoted CTC cluster formation by facilitating cell-cell adhesion.^245,246^ The role of chemokines, a group of signaling proteins that recruit immune cells to the location of inflammation or infection, in CTC cluster formation. CTCs secreted several chemokines, including CXCL1 and CXCL2, which promoted CTC cluster formation by attracting neutrophils, a type of immune cell, to the site of CTCs.^247,248^ The role of hypoxia, a condition of low oxygen levels, in CTC cluster formation. Hypoxia upregulated the expression of a transcription factor called HIF-1α, which promoted CTC cluster formation by upregulating EMT genes and downregulating cell adhesion molecules. The role of physical forces, such as shear stress and hydrodynamic forces, in CTC cluster formation. CTCs were more likely to form clusters in areas of low shear stress and high hydrodynamic forces, such as in the corners of blood vessels.^249–251^ In summary, CTC cluster formation is a multifactorial and complex process that entails multiple biological and physical factors. Despite our incomplete comprehension of the mechanism of CTC cluster formation, technological advancements have facilitated a more precise and comprehensive examination of CTCs and CTC clusters, offering new potential for understanding and treating metastatic cancer.

### Aniokis

When epithelial cells detach from their surrounding tissue, they usually undergo programmed cell death known as anoikis, which limits the efficacy of metastatic seeding.^72–74^ However, a small proportion of tumor- or metastasis-initiating cells with stem-like characteristics have been found to have an enhanced ability to survive and seed tumors.^47,75,85^ EMT is considered as a prerequisite for metastatic dissemination, as it enhances contact-independent survival and invasive potential.^78,79^ This indicates that EMT may function as a modulator rather than a promoter of metastasis. Meanwhile, a moderate level of EMT has been observed in CTCs, which links to stem-like properties, plasticity, reduced response to treatment and disease development.^76,85^ On the invasive edge of xenografts and patient tumor tissues of common carcinoma types, intermediate EMT stages have been seen.^84,86,87^ However, further investigation is needed to determine their functions in invasion, and metastatic dissemination.

The investigation of EMT within the context of CTCs is a field that is rapidly evolving, with numerous essential factors being explored. Commonly examined within CTCs are EMT-associated genes such as Vimentin, adhesion molecules, and core transcription factors. The analysis of signaling pathways triggered by membrane receptors and Receptor Tyrosine Kinases (RTKs) is a common practice in CTC studies, as these pathways, such as EGFR, TGF-β, Notch, c-Met, and Wnt, are crucial in the regulation of EMT. The investigation of Axl, an RTK associated with EMT, is gaining momentum in CTC studies, given that clinical trials are currently evaluating commercial inhibitors for Axl. It is noteworthy that EMT is associated with cancer-stem cells, and the majority of EMT-related markers examined in CTCs affect CTC survival and the ability to metastasize. Numerous studies have provided evidence of the heterogeneity of EMT-associated molecules present in the CTC population and have identified hybrid epithelial/mesenchymal (E/M) phenotypes in lung cancer. Table 2 highlights these findings.

**Table 2:** Summary of the clinical prognosis of CTC markers

EMT and Stemness Markers (+Other Associated)	Correlation between EMT Markers and Clinical Parameters	References
EpCAM, CK8, CK18, CK19, Vimentin, Twist, OCT4	Associated with distant metastasis and correlated with high total CTC counts	^72–74^
EpCAM, Vimentin	Poor response to chemotherapy and correlation with a decreased PFS	^47,47,75,75,85,85^
EpCAM, CK8, CK18, CK19, Vimentin, EGFR, KRAS	Reduced OS	^78,79^
EpCAM, CK8, CK18, CK19, Twist, Vimentin	Associated with metastasis	^72–74^
EpCAM, EGFR, CK7	Negative correlation with clinical stage	^74^
CK8, CK18, CK19, EpCAM, Vimentin, N-Cadherin, PD-L1	Associated with poorer survival	^85^
CK8, CK18, CK19, AXL, EGFR	Poor prognosis	^86^
PD-L1, EpCAM, CK8, CK18, CK19, Vimentin, Twist, PD-L1 EGFR, KRAS, BRAF, ROS1 mutation, ALK rearrangement	Poor prognosis	^86^
Vimentin, CK, AXL	Poor prognosis	^85^
EpCAM, Vimentin, CK	Poor prognosis	^85^

### Intravasation of CTCs

Intravasation, which involves the entry of cancer cells into the bloodstream, can occur through either active or passive mechanisms. The exact mechanism utilized by the tumor cells depends on various factors, including the tumor types, the micro-environment, and the integrity of the blood vessels.^252^ Active intravasation occurs when tumor cells or clusters actively migrate into the bloodstream through EMT. Passive intravasation, on the other hand, happens when single cells or groups of cells separate from the main promise and enter the bloodstream as a result of broken tumor blood vessels brought on by tumor growth or treatment.^181^ After entering the bloodstream, CTCs are subjected to various obstacles that can threaten their survival, including shear stress caused by blood flow, anoikis, all of which can potentially lead to their elimination.^253^ Tumor cells can gain access to the bloodstream through either a blood vessel or a lymphatic vessel, which can be influenced by several factors such as accessibility, physical constraints, and the existence of active mechanisms that lure cells to specific types of vessels.^254,255^ Additionally, tumor cells can utilize lymphatic vessels as a pathway to enter the bloodstream since these vessels ultimately empty into the major thoracic duct.^254,256^ Although lymphatic vessels can aid in the entry of certain cancer cells into the blood circulatory system, current evidence is limited, and cancer cells may encounter dead ends in lymphatic deposits. This observation highlights the degree of concurrent dissemination from the primary tumor.^75,257,258^ Alternatively, cancer cells are likely to spread primarily through the bloodstream for distant metastasis. In this process, lymphatic fluid passes through a sequence of lymph nodes, which frequently serve as the primary sites of metastasis.^252,259^

### Colonization of CTCs

The colonization of tumor cells is the final step of the metastasis process and involves the establishment of tumor cells in a distant organ.^232,260,261^ The colonization process is complex and involves several steps that are regulated by multiple signaling pathways and molecular networks. This process requires the re-activation of epithelial factors and the suppression of mesenchymal ones, a process known as MET. Several studies have investigated the molecular mechanisms underlying this process in various cancer types. One of the key players in the colonization of tumor cells is p63, which is a member of the p53 group of tumor suppressors. P63 is upregulated during MET in a variety of cancer types and is essential for the maintenance of epithelial stem cells.^262^ P63 promotes the reactivation of epithelial genes like E-cadherin and cytokeratins, while suppressing mesenchymal genes like Vimentin and fibronectin.^263^ Twist and Snail have also been implicated in the regulation of MET and the colonization of tumor cells. Twist is upregulated during EMT and downregulated during MET.^264^ Twist improves the mesenchymal phenotype of tumor cells by suppressing E-cadherin expression and increasing N-cadherin and fibronectin expression.^265^ Snail, on the other hand, promotes EMT and invasion by inhibiting E-cadherin expression, but its role in MET and colonization is less clear. Several signaling pathways also participated in the regulation of MET and tumor cell the colonization.^266,267^ The TGF-β pathway, for example, can induce EMT in various cancer types but can also promote MET and colonization by activating the expression of p63 and other epithelial genes.^268^ Other signaling pathways, such as Notch, Hedgehog, and Wnt, have also been implicated in the regulation of MET and colonization in various cancer types.^269,270^ During metastasis, CTCs and the target organ microenvironment engage in a critical dialog that determines the fate of metastatic colonization. This interaction encompasses various components such as ECM proteins, immune cells, and soluble factors, which collectively influence the survival, dormancy, and growth of CTCs. Key processes include the EMT, which CTCs undergo to establish new tumors, and the evasion of immune surveillance. The microenvironment’s role extends to either supporting CTC colonization through the provision of growth factors or hindering it by presenting physical barriers and immune challenges.^271,272^

## The CTCs under immune system surveillance

**Fig. 4:**
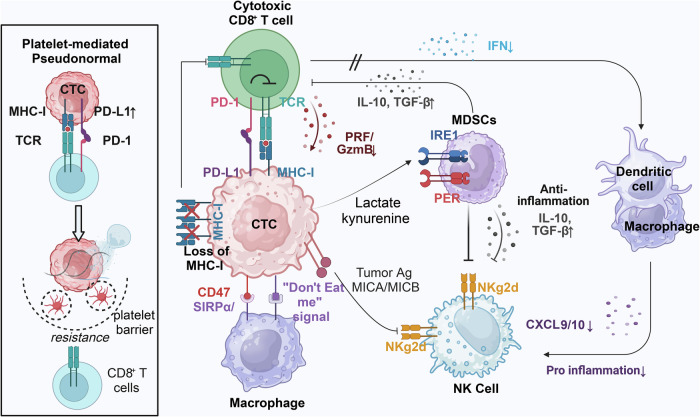
Immune-escape mechanisms of CTCs in the peripheral blood. The diagram depicts the various mechanisms employed by CTCs to circumvent the immune system and the interactions that take place between CTCs and immune cells in the peripheral blood. The interaction between circulating tumor cells (CTCs) and natural killer (NK) cells is a prominent area of investigation, given that CTCs secrete LDH5 and shed the MICA/MICB ligand via ADAM10, which inhibits recognition and elimination of CTCs through NK cell-mediated lysis. The diagram portrays three mechanisms employed by CTCs to evade recognition by NK cells and T cells through MHC I molecules. These strategies include masking MHC I recognition by TCR via cytokeratins (CK8, CD18, and CK19) bound to the cell surface, gaining a “pseudo-normal” phenotype through membrane transfer from platelets to CTCs, and reducing or eliminating MHC I expression altogether. The interaction between CTCs and natural killer (NK) cells is a key area of focus, as the release of LDH5 and shedding of MICA/MICB ligands by ADAM10 from CTCs prevents their recognition and elimination via NK cell-mediated lysis. Additionally, LDH5 enhances NKG2D ligand expression on circulating monocytes, which in turn reduces NKG2D expression on NK cells. Furthermore, the diagram illustrates other strategies employed by CTCs to evade the immune system, such as the upregulation of the inhibitory immune checkpoint molecule PD-L1, expression of the “don’t eat me” signaling receptor CD47 and altered expression of apoptotic proteins FAS and/or FASL. The figure was created with BioRender.com

The mechanism by which CTCs overcome immune system surveillance is not completely understood, but there are several possible ways in which they may evade detection by the immune system. One explanation is that CTCs suppress or mask the expression of surface antigens that immune cells often recognize as abnormal or foreign. This can make the CTCs less visible to the immune system and allow them to escape detection. Another possibility is that CTCs produce factors that suppress or inhibit the immune response, such as cytokines or chemokines that recruit immune cells to the tumor microenvironment but then suppress their function. For example, studies have shown that CTCs can produce the cytokine TGF-β, which can suppress the activity of T cells and other immune cells (Fig. 4). Finally, CTCs may be able to evade immune surveillance by mimicking normal cells or using other mechanisms to avoid detection. For example, some CTCs may express proteins or molecules that are normally present in healthy cells, leading to a challenge for the immune system in differentiating them from healthy cells.^273,274^

### Immune evasion through antigen loss

CTCs can downregulate or lose expression of antigens normally recognized by the immune system, making them less visible to immune cells. MHC class I molecules, for example, are required for T cell recognition, and their expression is downregulated by certain CTCs. For example, in 2012, researchers found that the expression of EpCAM, a common antigen used for CTC detection, was downregulated in CTCs from breast cancer patients who had received chemotherapy.^275,276^ Similarly, a study reported that CTCs from breast patients who received anti-HER-2 therapy reduced their expression of HER-2, another common antigen.^277–279^

### CTCs secreted immunosuppressive factors

CTCs have the ability to secrete immunosuppressive factors including TGF-β and IL-10, which can inhibit immune cell activation and function and create an immunosuppressive milieu that shields CTCs from immune attack. A previous work demonstrated that CTCs from breast cancer patients secrete high levels of TGF-β, which can and promote tumor growth and metastasis because of immune cell inactivation.^280–282^ Similarly, another study showed that CTCs from melanoma patients release IL-1, which has the ability inhibit T cell activation and proliferation, a crucial part of the immune system’s defense against cancer.^103,283,284^ Besides, Zhao et al. reported that CTCs from lung cancer patients secrete CCL2, which can attract immune-suppressive cells to the tumor microenvironment, thereby suppressing the immune response. Then, a clinical trial demonstrated that CTCs from prostate cancer patients secrete GAL-3, which can induce the apoptosis of tumor-killing immune cells, thereby blocking the immune response against cancer.^285–287^ In order to promote angiogenesis and suppress the immune response, CTCs from breast cancer patient have also been reported to secrete VEGF.^288–290^ Also, CTCs from colorectal cancer patients secreted PGE2 and led to immune suppression and tumor progression as well.^291,292^ Moreover, CTCs from melanoma patients secrete IL-10, which can inhibit the proliferation and promote tumor growth and metastasis by activating T cells.^293,294^ A study showed that CTCs from lung cancer patients expressed high levels of programmed cell death ligand 1(PD-L1), which inhibited the activity of immune cells and promoted immune evasion.^295–298^

### Immunological checkpoint (IC) proteins

CTCs have the ability to express IC proteins, such as PD-L1, which bind to the receptors on immune cells to prevent the immune cells from activating and functioning. This can prevent immune cells from attacking CTCs and allow them to escape immune surveillance. CTCs from patients with advanced colorectal, breast, or prostate cancer exhibit IC proteins such PD-L1 and CTLA-4, which can promote immune evasion and support in tumor growth, according to Krebs MG.^297–299^ Maheswaran et al. demonstrated that CTCs from lung cancer patients express PD-L1 and other IC proteins, and that PD-L1 expression is linked to poor prognosis.^300,301^ Gkountela et al. reported that CTCs from breast cancer patients express PD-L1, which is linked to resistance to chemotherapy.^302^ Zhang W et al. showed that CTCs from gastric cancer patients express PD-L1 and TIM-3, which is associated with advanced stage and poor prognosis.^303^ Sanmamed et al. demonstrated that CTCs from melanoma patients express PD-L1 and other IC proteins, and that PD-L1 expression leads to treatment resistance and poor outcome.^304^ Similar results have been observed in patients with breast cancer.^305^ Another study revealed that CTCs from lung cancer patients who received IC inhibitor therapy have downregulated PD-L1 expression.^306–308^ The dual inhibition strategy of blocking PD-L1 and CD47 on CTCs has been found to significantly enhance the effectiveness of IC therapy. By enabling the immune system to more efficiently target and eliminate CTCs, this approach holds promise for reducing the risk of tumor recurrence and metastasis. Preclinical models have demonstrated that this combination therapy is more effective, indicating its potential for successful clinical application in cancer treatment.^309^

### Production of extracellular vesicles

By promoting the detachment of CTCs from the original site, extracellular vesicles (EVs) play a vital role in the metastatic process. EVs enhance the shedding of CTCs and protect them in circulation, thereby influencing their metastatic direction. The detachment of CTCs from the main site is facilitated by EVs, which also contribute to EMT and ECM remodeling, promote angiogenesis, and improve vascular permeability. In addition, EVs protect CTCs by activating platelets, inducing immunosuppression, and determining the organotropism of metastasis, thus influencing the colonization of CTCs in distant organs.^310^ EVs released by CTCs can interact with immune cells and modulate their function, creating an environment that favors CTC survival and growth. Armstrong AJ showed that CTCs from prostate cancer patients produce extracellular vesicles that can suppress the immune response by suppressing the T cell and NK cell activity.^311–313^ Keklikoglou demonstrated that CTCs from breast cancer patients produce extracellular vesicles that can promote immune evasion by inducing the differentiation of monocytes into immunosuppressive macrophages.^314–316^ Whiteside TL et al. discussed the role of extracellular vesicles produced by CTCs and other tumor cells in promoting immune suppression and tumor progression.^317^ Peinado H et al. showed that CTCs from breast cancer patients produce extracellular vesicles that can promote metastasis by modifying the microenvironment at distant sites and suppressing the immune response.^318^ Liu C et al. demonstrated that extracellular vesicles produced by CTCs from lung cancer patients can induce immunosuppression by blocking the activity of NK and T cells.^319^ Melo SA et al. showed that CTCs from pancreatic cancer patients produce extracellular vesicles that can promote immune evasion by inducing the differentiation of monocytes into immunosuppressive macrophages.^320^ Lu J et al. demonstrated that extracellular vesicles produced by CTCs from lung cancer patients can induce immunosuppression.^321,322^ Hoshino et al. showed that CTCs from melanoma patients produce extracellular vesicles that can promote immune evasion by inhibiting the activity of tumor-killing immune cells, and that targeting these vesicles can enhance the effectiveness of immunotherapy.^323^

### Formation of immune-resistant micro-metastases

CTCs can establish small metastatic lesions that are immune-resistant. These lesions can create a reservoir of CTCs that can escape immune surveillance and facilitate further metastatic spread. ICAM1 is essential for the development of CTC clusters and their trans-endothelial migration in lung metastases of BRCA. It has been found to facilitate metastasis more effectively in clusters as opposed to single cells, thereby contributing to a decrease in overall survival rates. In lung metastases of patient-derived xenografts of TNBC, the expression of ICAM1 is noticeably elevated. The inhibition of ICAM1 has been shown to impede the lung colonization of TNBC cells by disrupting the formation of tumor cell clusters, suggesting that ICAM1 could serve as a promising therapeutic target for inhibiting metastasis initiation in TNBC.^100^ Janssen LME showed that CTCs can form immune-resistant micro-metastases by secreting extracellular vesicles that modulate the immune response and create a favorable microenvironment for metastatic growth.^324^ Krebs et al. demonstrated that CTCs can form micro-metastases that are resistant to chemotherapy and immune surveillance in breast cancer patients.^325^ Focusing on the formation of osimertinib-resistant micro-metastases following treatment, a study described an orthotopic model of lung cancer. This model provides a platform for analyzing resistance mechanisms and evaluating new therapeutic strategies against metastases of NSCLC.^326^ Kallergi et al. showed that CTCs can form micro-metastases that are resistant to chemotherapy and immune surveillance in breast cancer patients, and that targeting the immune system can improve treatment outcomes.^326^ Aceto N et al. showed that CTCs can form micro-metastases that are resistant to chemotherapy and immune surveillance in breast cancer patients, and that targeting the immune system can improve treatment outcomes.^327^ Bidard FC et al. demonstrated that CTCs can form micro-metastases that are resistant to chemotherapy and immune surveillance in breast cancer patients, and that targeting the immune system can improve treatment outcomes.^328^

### Platelets coordinate with CTCs

It has been indicated that the lifespan of certain CTCs is brief, as a majority of them are undetectable within 24 h after primary tumor excision.^329^ Research reported that the interaction of CTCs with other blood components, particularly platelets, significantly affects their survival and potential for metastasis.^329^ CTCs shortly after entering the bloodstream, are known to create a thrombus rich in platelets around them, serving as a protective shield against shear stress and immune response. Furthermore, this thrombus promotes the attachment of CTCs to the endothelial lining of blood vessels, enabling extravasation.^121,309,330^ Upon entering the bloodstream, CTCs can form a shield of platelets through the action of platelet tissue factor. The formation of a thrombus rich in platelets can offer protection to CTCs against shear stress and immune system attacks, while also aiding in the attachment of tumor cells to the blood vessel wall and extravasation. Moreover, studies have shown that activated platelets can transfer the MHC to CTCs, enabling them to evade immune surveillance by mimicking host cells.^117,331^ In addition, platelets have been shown to reduce the recognition and elimination of tumor cells by NK cells. Platelets can release soluble factors that impair the cytotoxicity of NK cells and promote the expansion of regulatory T cells, which further interfere with the immune responses. Platelets can also inhibit the recognition and destruction of tumor cells by immune cells, as they express molecules that interact with receptors on immune cells and prevent their activation. One such molecule is CD47, which can bind to SIRPα on macrophages and inhibit their engulfment of tumor cells. These mechanisms collectively enhance the survival and dissemination of CTCs.^117,243^

Moreover, the transfer of MHC molecules by platelets is a mechanism by which CTCs can evade immune surveillance.^117,332^ Platelets have the ability to impede the identification and destruction of neoplastic cells by NK cells.^18,322,333^ The invasiveness and metastasis of CTCs can be enhanced by platelets, which release TGFβ and promote the EMT in these cells. This phenomenon has been demonstrated in previous research.^107,334^ CTCs have been found to interact with different types of leukocytes such as, monocytes, and macrophages, which can enhance the survival of CTCs and facilitate their interaction with endothelial cells, leading to extravasation.^21,107^

## Role of epigenetic modifications in deciphering the properties of CTCs

Recent research has highlighted that significant epigenetic alterations in normal cells can lead to the acquisition of a malignant phenotype.^335^ Epigenetics involves the investigation of inheritable alterations in gene expression that occur without modifications to the DNA sequence itself.^336^ A number of epigenetic modifications, including histone acetylation and methylation patterns, microRNA-mediated gene regulation, hypomethylation of oncogenes, hypermethylation of tumor suppressor genes, and others, greatly contribute to the progression of cancer.^337^ Understanding the intratumor heterogeneity and gaining insight into tumor-specific epigenetic markers linked to metastasis may be facilitated by mapping the epigenetic landscape of CTCs. This approach may also identify subpopulations of CTCs capable of metastatic dissemination. The main areas of epigenetic research that apply to this scenario are gene regulation and DNA methylation.

### DNA methylation

DNA methylation is the addition of methyl groups to DNA’s cytosine residues, and it is essential for regulating gene expression. DNA methylation patterns that deviate from the normal or expected patterns have been linked to various health conditions, including cancer. Several studies have shown that CTCs have distinct DNA methylation profiles compared to primary tumors, which could be useful in detecting early-stage cancer, predicting cancer prognosis, and developing targeted therapies.^338,339^

CTCs exhibit unique DNA methylation patterns that differ from primary tumor.^339^ For instance, in breast cancer patients, CTCs had increased DNA methylation levels compared to primary tumors.^121^ They identified several differentially methylated genes that were specific to CTCs, including the FBLN1, FBN2, and IGFBP6 genes. A prognostic factor for cancer may be the DNA methylation status of CTCs, as these genes have been linked to the progression and metastasis of cancer. Similarly, in prostate cancer, CTCs had unique DNA methylation patterns compared to primary tumors.^340^ They identified several differentially methylated genes, including the FOXA2, HOXA9, and PTEN genes. These genes are involved in cancer progression, invasiveness, and metastasis. Additionally, they discovered a correlation between the DNA methylation status of CTCs and patient survival, suggesting that this biomarker may have applications in the prediction of cancer prognosis. Moreover, DNA methylation patterns of CTCs can also differ based on the origin of the tumor. For instance, a study by Xu et al. compared DNA methylation profiles of CTCs separated from lung cancer and pancreatic cancer patients.^341^ They found that CTCs from lung cancer patients exhibited higher DNA methylation levels compared to CTCs from PAAD patients. Furthermore, they identified differentially methylated genes specific to each cancer type, such as the RASSF1A gene in lung cancer CTCs and the TGFBI gene in pancreatic cancer CTCs. This evidence support that the DNA methylation pattern of CTCs could be used to distinguish between different cancer types and aid in personalized cancer treatment.^342^

In addition, DNA methylation patterns of CTCs can also be influenced by treatment. DNA methylation patterns of CTCs changed after chemotherapy in breast cancer patients.^343^ They identified several genes, including the CDH1 and ZEB1 genes, which had differentially methylated CpG sites before and after chemotherapy. These results imply that monitoring alterations in the DNA methylation patterns of CTCs could be utilized to evaluate the effectiveness of cancer treatments.

Further research is required to unravel the gene methylation landscape of CTCs and how these methylation changes contribute to CTC-mediated metastasis. Such studies can reveal the tumor-specific epigenetic characteristics related to metastasis and the heterogeneity of tumor populations.^344,345^ Gkountela et al. analyzed the DNA methylation patterns of CTCs from BRCA patients and tumor xenograft models in NSG mice and discovered numerous differentially methylated regions (DMRs) in CTCs.^5,346^ CTCs showed lower DNA methylation levels of genes such as JUN, MIXL1, SHOX2, and MEF2C, which are often enriched in various types of cancer. CTC clustering was associated with low methylation levels of TFBSs that regulate and proliferation- and stemness-related genes, such as OCT4, MANOG, SOX2, and SIN3A.^17,97,347^ This was accompanied by hypermethylation and H3K27me3 repression of target gene promoters and bodies of PRC2 targets, including SUZ12 and EED, collectively increasing the hematogenous metastatic potential of CTC clusters.^348,349^ In a study comparing the DNA methylation of CTCs from cancer patients and normal blood controls, abnormal methylation changes were found in multiple genes, and were associated with resistance to sunitinib. This ample evidence suggests that abnormal gene methylation in CTCs may play a crucial role in their clustering and promote distant metastatic ability (Table 3).

**Table 3:** Summarizes the aberrant methylation of different genes in CTCs across cancers

Cancer type	Model of CTC studied	Genes studied	Functional class of the genes	Nature of methylation change	Effect of aberrant methylation on cancer cell	Reference
Breast cancer	Patient-derived CTCs	*SOX17* *CST6*	tumor suppressor	hypermethylated	promotes invasion	^5^
		*BRMS1*	tumor suppressor	hypermethylated	promotes invasion	^5^
		*ESR1*	metastasis suppressor	hypermethylated	promotes invasion, stemness, and mesentchymal phenotype	^5^
		*ITIH5*			promotes tumor growth and resistance to immunotherapy	^346^
		*RASSF1A*	tumor suppressor	hypermethylated	increased invasiveness, stemness, and proliferative potential	^97^
		*NANOG*	tumor suppressor	hypermethylated	increased invasiveness, stemness, and proliferative potential	^5^
		*SOX2*	tumor suppresor	hypermethylated	promotes cluster formation in CTCs	^97^
		*SIN3A*	represses oncogenic activity	hypomethylated	promotescluster formation in CTCs	^5^
		*RASSF1A*	transcription factor that helps maintain self-renewal and pluripotency		promotes cluster formation in CTCs	^346^
		*hVIM*	represses oncogenic activity	hypomethylated	increases metastatic potential of CTCs	^347^
Melanoma	Patient-derived CTCs	*SFRP2*	tumor suppressor	hypermethylated	increases invasion	
Colorectal Cancer	Patient-derived CTCs	*RASSF1A*	tumor suppressor	hypermethylated	increases metastatic potential	
		*MLL3*	tumor suppressor	hypermethylated	promotes proliferation	
		CDH1	EMT suppressor	hypermethylated	activates EMT and increases invasiveness	

#### Aberrant DNA methylation leading to mutation and inactivation of oncogenes or tumor suppressor genes

TSGs play a critical role in orchestrating cellular processes and maintaining genomic stability. Their mutations can cause excessive cell proliferation, which can contribute to the development of tumors. They are also in responsible for causing apoptosis, repairing DNA damage, and regulating cell division. One example of a TSG is hMLH1, which is involved in DNA mismatch repair.^350^ Studies have shown that hMLH1 gene mutations and Microsatellite Instability are positively correlated with hMLH1 promoter hypermethylation. The Knudson hypothesis proposes that mutations in both alleles are necessary to inactivate TSGs, and epigenetic modifications, such as the silencing of one allele through promoter hypermethylation, can satisfy this requirement.^351^

**Fig. 5:**
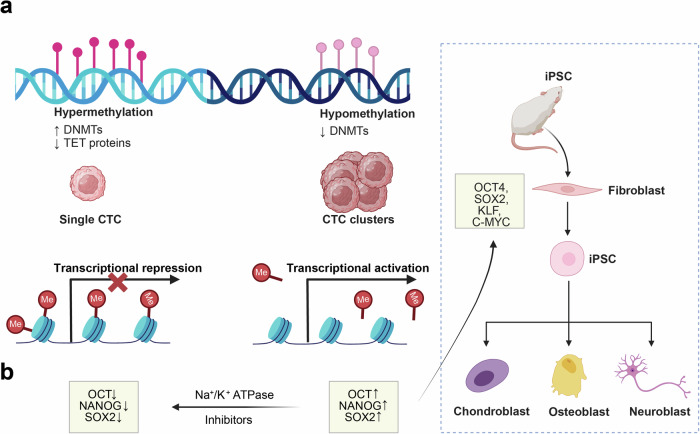
Different DNA methylation modifications operating in CTCs to promote metastasis. The process by which methylation promotes detachment and invasiveness of CTCs is intricate and involves both hypermethylation and hypomethylation of various genes. **a** Specifically, hypermethylation of tumor suppressor genes (TSGs) and metastasis-associated genes (MSGs) triggers the detachment of CTCs and contributes to their enhanced proliferative capacity. CTC clusters exhibit distinct DNA methylation profiles compared to single CTCs, featuring hypomethylation of binding sites for transcription factors like OCT4, NANOG, SOX2, and SIN3A, **b** which are stemness-related transcription factors play crucial roles in the pluripotency network of induced pluripotent stem cells (iPSCs). The figure was created with BioRender.com

Some patients showed evidence of epigenetic silencing of the MGMT gene by promoter hypermethylation, which was connected to a higher frequency of activating mutations in KRAS, a proto-oncogene frequently mutated in colorectal cancer. It has been indicated that hypermethylation of the MGMT promoter may contribute to the development of colorectal cancer by promoting KRAS mutations through the inactivation of a DNA repair gene. A study of 244 patients with colorectal cancer analyzed the effects of epigenetic alterations on proto-oncogenes by examining the MGMT gene. The study found that the promoter hypermethylation-mediated MGMT inactivation was linked with a rise in guanine-to-adenine mutations in K-Ras oncogenes. The MGMT gene expression helps to prevent such transitions in the Ras genes.^352^ The aforementioned discoveries underscore the essential function of epigenetic modifications in stimulating proto-oncogenes and consequently, advancing the growth and advancement of cancer. A study revealed that lung cancer CTCs demonstrate a distinctive DNA methylation pattern in comparison to primary tumors and normal tissues. This signature is characterized by a substantial reduction in CTC DNA methylation overall, indicating a process of gradual demethylation that progresses from primary tumors to normal tissues and ultimately to CTCs. This underscores the dynamic epigenetic alterations linked to cancer metastasis and spread. Notably, this demethylation phenomenon is observed throughout various genomic regions, encompassing promoters, gene bodies, introns, and intergenic regions. There is a noticeable decrease in methylation at CpG-poor promoters in CTCs when contrasted with primary tumors.^338^ We have summarized the different mechanisms of DNA methylation modifications in CTCs to promote metastasis in Fig. 5.

#### Hypomethylation regulation of CTC clusters and increases metastatic potential

The sustained proliferation and self-renewal capacities of embryonic stem cells require the maintenance of stemness and proliferation properties.^353^ It has been indicated that stemness-related genes may be entailed in the dissemination of cancer cells. However, the precise mechanisms underlying the ability of CTCs to disseminate and form metastases are not yet fully elucidated, and their survival in the bloodstream is critical for this process. A research study on breast cancer patients aimed to examine the metastasis of CTCs by investigating their ability to form clusters. In the study, single CTCs and CTC clusters from patients with progressing breast cancer were isolated using Parsortix microfluidic technology. Subsequently, whole-genome bisulfite sequencing was conducted on single-cell resolution matched CTCs, which were selected from eight out of a total of 43 analyzed samples (19%). The sequencing outcomes revealed that clusters had hypomethylation of stemness-related genes, specifically SOX2, NANOG, and SIN3A, compared to sing CTCs. This observation facilitated the identification of potential therapeutic targets that might diminish the metastatic potential of the CTC clusters. Specifically, the researchers identified the alteration of methylation patterns in the promoter of the related genes by a Na^+^/K^+^ ATPase inhibitor led to the separation of the clusters and the reduced tumor metastasis in mice.^5^

A study identified DMRs between CTCs and primary lung cancer samples, revealing a significant number of hypomethylated DMRs in CTCs. This trend towards decreased DNA methylation at aberrantly methylated loci in CTCs suggests a potential mechanism underlying their unique characteristics. A potential function in regulating crucial physiological processes such cell proliferation, differentiation, and apoptosis was discovered in the hypomethylated regions of CTCs, which were found to be highly enriched for transcription factor binding sites (TFBSs). Consequently, the evidence supports the idea that hypomethylation in CTCs may play a crucial role in the formation of CTC clusters and enhance their metastatic potential by influencing gene expression related to EMT and other pathways critical for cancer progression.^338^ The significance of this study lies in its use of genome-wide sequencing-based methylation profiling for CTCs, making it the first of its kind. This emphasizes the importance of generating comprehensive methylation patterns for CTCs in the context of broader biological research. The incorporation of advanced technology in this research signifies a significant advancement in the current understanding of metastasis and the recognition of novel drug targets.

#### Hypermethylation regulation the invasiveness of CTCs

The process of detachment of CTCs from their primary tumors and their subsequent migration to distant organs is a pivotal event in the progression of metastasis. To facilitate this process, the cells require the ability to detach and enhance their plasticity, allowing them to penetrate through capillaries and settle in different organs. The mechanism that plays a crucial role in this process is known as EMT, which endows the cells with increased invasiveness and motility.^354^ Utilizing peripheral blood samples from 52 patients with metastatic colorectal cancer, the researchers extracted CTCs and examined the methylation patterns of two crucial EMT genes, Vimentin and SFRP2, to investigate the function of EMT in CTC invasiveness. The findings of the study indicated that the selected genes were heavily methylated in the isolated CTCs as compared with the healthy tissue samples. The study demonstrated that the Vimentin gene was suppressed by DNA methylation, causing a disruption in the formation of the cytoskeleton, and consequently promoting increased plasticity and invasiveness of the cells. In contrast, the study revealed that the suppression of the SFRP2 gene by methylation activated the Wnt signaling pathway, which led to increased invasiveness of the CTCs.^355^ In another study, researchers evaluated the methylation status of E-cadherin, an EMT suppressor gene that is responsible for preserving the epithelial phenotype. The methylation pattern was assessed in CTCs obtained from six individuals diagnosed with metastatic prostate cancer. The findings indicated that E-cadherin was highly methylated in the CTCs from the cancer patients. Furthermore, the study presented an association between E-cadherin hypermethylation and an increased invasiveness of CTCs.^356^ The role of EMT in CTCs is crucial for their adaptability and survival in the bloodstream during the early stages of metastatic colonization, as highlighted in a recent review. Therefore, a comprehensive analysis of EMT in CTCs is essential for informing personalized medicine strategies that target specific aspects of this biological process.^178^ The research conducted by Zavridou et al. offers compelling evidence for the effectiveness of the size-based method in addressing the heterogeneity of CTCs.^357^

#### DNA methylation and CTCs immune escape

**Fig. 6:**
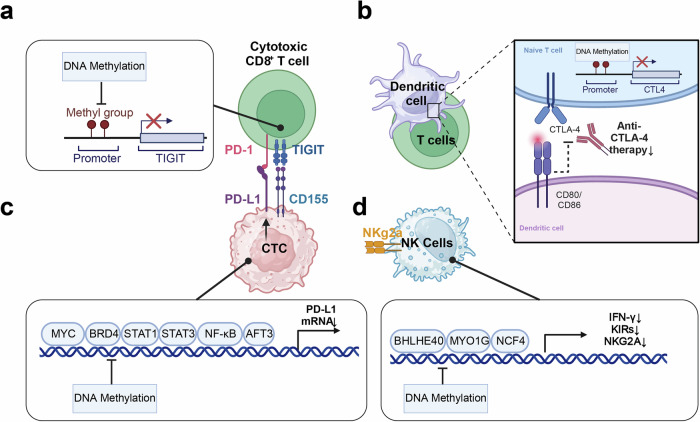
DNA methylation regulation in CTCs related Immunity. DNA methylation play key roles in adaptive immune response, including dendritic cell development and T cell priming and activation. **a** Recent studies revealed the contributions of chromatin remodeling responding to cytotoxic attack in tumor cells and exhaustion phenotype in tumor infiltrating CD8^+^ T cells. **b** CTLA-4 DNA hypermethylation significantly correlated with a poor response to treatment, highlighting the potential of CTLA-4 methylation as a predictive biomarker for therapy outcomes. **c** In tumor cells, DNA modifications affects production of tumor antigens, silencing of anti-tumor cytokines, and induction of the PD-L1 checkpoint. **d** In NK cells, the methylation status of killer Ig-like receptors (KIR) CpG islands is crucial for maintaining clonal KIR expression and modulating NK cell recognition and lysis of abnormal cells. The figure was created with BioRender.com

The dysregulation of DNMTs and TET enzymes can profoundly influence gene expression and contribute to transcriptional silencing or activation in various pathologies, such as cancer.^358^ Studies have provided evidence indicating that the dysregulation of TET enzymes and DNMTs can have significant impacts on gene expression, leading to transcriptional activation or silencing in several pathological states, including cancer. Studies have shown that breast cancer and colorectal cancer patients exhibit increased expression of TET enzymes and decreased expression of DNMTs in both their tumor tissues and circulation, which correlates with DNA hypomethylation and upregulation of IC molecules or their ligands^359,360^ (Fig. 6).

Furthermore, it has been demonstrated that restoring normal DNA methylation patterns can enhance the therapeutic efficacy of various cancer treatments. Specifically, the use of epigenetic modifiers that target DNA methylation in combination with chemotherapy, radiotherapy, and immunotherapy has shown increased treatment effect on preclinical models and early clinical trials of several cancers, including breast, prostate, and colon cancer.^361–363^ Based on the outcomes of diverse preclinical models and preliminary clinical trials, it appears that epigenetic modulators aimed at DNA methylation may have the ability to function as adjunctive therapy to conventional cancer treatments. Nevertheless, further large-scale clinical trials are required to verify these findings and establish the optimal combination of treatments for different cancer types. To sum up, there is a correlation between abnormal DNA methylation patterns in cancer cells and immune evasion, tumorigenesis, and resistance to cancer treatments. Therefore, DNA methylation patterns may be a crucial target in cancer therapy, and reinstating regular DNA methylation patterns could be a promising strategy to enhance the therapeutic efficacy and overcome resistance mechanisms.

The evidence provided by these studies suggests that the control of ICs and IC ligand expression in cancer patients, particularly in breast and colorectal cancers, is critically influenced by DNA methylation. According to the study, significant upregulation of ICs, including PD-L1 and TIGIT, as well as other ICs/IC ligands in both circulation and tumor tissues, can be attributed to DNA hypomethylation. The significance of DNA methylation in predicting the effectiveness of immunotherapeutic treatments is highlighted by the correlation between overall hypomethylation and inadequate clinical responses. Based on these findings, therapeutic manipulation of DNA methylation has the potential to serve as an effective strategy for enhancing the outcomes of immunotherapies. However, further investigation is demanded to fully understand the mechanism by which DNA methylation impacts the expression of ICs and to develop efficient therapeutic approaches that target these epigenetic alterations.^364^

DNA methylation alterations have been linked to the expression of several immunomodulatory genes in breast and colorectal cancer tissues. A study demonstrated that the hypomethylation of CpG islands led to the upregulation of PD-1, CTLA-4, and TIM-3 in breast cancer.^359^ Moreover, the study observed complete hypomethylation of LAG-3 gene promoter regions in both breast tumor tissues and their corresponding normal tissue. This suggests that DNA methylation is not entailed in the elevation of these genes in breast cancer.^359^ According to another study, DNA hypomethylation is responsible for the overexpression of CTLA-4 and TIGIT in human colorectal cancer tissues.^360^ By contrast, in colorectal tumor tissues, the PD-1, PD-L1, galectin-9, and TIM-3 overexpression was not found to be associated with DNA methylation, according to the study.^360^ Marwitz et al. reported that the PD-1 and CTLA-4 upregulation in tumor tissues of patients with lung cancer is attributed to DNA hypomethylation.^365^ However, the study did not find any relationship between the elevated PD-L1 expression and DNA methylation in the tumor tissues.^365^ Conversely, DNA hypomethylation was discovered to be the underlying cause of the upregulation of PD-L1 expression in HNSCC tumor tissues.^366^ In a study by Goltz et al., the promoter methylation of PD-L1 was predictive of favorable prognosis in several cancer types, including colorectal cancer, HNSCC, and acute myeloid leukemia.^367,368^ DNA hypomethylation was identified as the cause of the increased expression of CTLA-4, PD-1, PD-L1, and PD-L2 in patients with lower-grade gliomas, according to a study by Rover et al. Overall, these findings support that DNA hypomethylation is entailed in the upregulation of IC molecules and ligands in different types of cancers, although the specific genes that are regulated by DNA methylation may vary across different cancer types.

#### DNA methylation of CTCs cluster formation

Recent research has suggested that there may be a relationship between CTC cluster formation and DNA methylation. One study found that DNA methylation changes in CTCs may contribute to the CTC cluster formation, and that these changes may be associated with increased metastatic potential. Specifically, the researchers found that methylation changes in genes associated with cell adhesion and motility were more frequent in CTC clusters than in single CTCs. Jang et al. investigates the DNA methylation status of CTCs in gastric cancer patients and its correlation with CTC cluster formation.^369^ The authors find that DNA methylation alterations in CTCs are associated with increased cluster formation and suggest that targeting these alterations may be a potential therapeutic approach.^370–372^ Gkountela et al. demonstrates that CTC clustering induces DNA methylation changes that promote metastatic seeding. The authors claim that targeting these epigenetic changes may be a potential therapeutic strategy for preventing metastasis.^373,374^ Zhang et al. demonstrate that DNA methylation changes in CTCs are involved in their ability to form brain metastases in breast cancer patients. The authors suggest that targeting these epigenetic alterations may represent a potential strategy for preventing brain metastases.^376^ Huang et al. investigates the correlation between DNA methylation in CTCs and their ability to resist EGFR inhibitors in lung adenocarcinoma patients.^253^ The findings of the authors indicate that targeting DNA methylation abnormalities in CTCs may enhance treatment response, as these variations are linked to higher resistance to EGFR inhibitors.^375,376^ Overall, while the relationship between CTC cluster formation and DNA methylation is still not fully understood, the current evidence suggests that changes in DNA methylation may be involved in the progression of metastatic cancer.

### Histone modification

Histones are essential components of the dynamic architecture of chromatin. The octameric core of these proteins is formed by the assembly of two copies of each histone variation, including H3, H4, H2A, and H2B. This structure serves as a pool around which a DNA sequence consisting of 146 base pairs coils elaborately.^377^ The globular structures of histones are enriched in basic amino acids like arginine and lysine in their tails, which serve as excellent locations for a wide range of covalent posttranslational modifications (PTMs). The chromatin landscape is altered by these chemical modifications, which also generate docking sites for proteins that control chromatin functionality.^378^ These modifications also modify the interaction between histones and DNA.^379^ Extensive studies has shown phosphorylation, methylation, and acetylation as the central histone modifications. All of these PTMs, however, add to the complexity of the regulation of gene expression by expanding the histone code. These additional PTMs include crotonylation, lactylation, citrullination, ubiquitination, and adenosine diphosphate (ADP)-ribosylation.^380^ Enzymes known as “writers”, “readers”, and “erasers”, whose dysregulation is usually linked to cancer, meticulously control this dynamic epigenetic gene landscape.^381^ Decoding the histone modification patterns presents a challenge since, even in identical cellular contexts, same configurations can produce divergent biological responses.^378^ Abnormalities in this molecular communication have the potential to cause oncogenic transformation,^382^ alter gene regulatory networks, and upset cellular equilibrium. Understanding this molecular dialog is critical for both understanding and fighting the molecular bases of CTC metastasis. It is also fundamental to elucidating cellular physiology.

#### Dysregulation in the landscape of histone modifications on TSG and oncogenes

By regulating transcriptional activity, histone alterations play a critical role in the initiation and proliferation of tumors. This often results in the upregulation of oncogenes and the downregulation of tumor suppressor genes. The erratic patterns of H3K27me3, which have a substantial influence on genomic stability, are a notable example of this dysregulation385.^383^ The enhancer of EZH2 gene, which encodes the methyltransferase responsible for the specific histone modification H3K27me3, can be influenced by recurring mutations that may alter the levels of this modification.^384^ The transcriptional landscape undergoes a significant reconfiguration during tumorigenesis. After being found to be tumor suppressors in a variety of cancers,^385^ CBP/p300 has lately come to light as essential regulators of transcriptional activation mediated by enhancers and super-enhancers, especially with regard to important oncogenes.^386,387^ In ALL, binding sites for the MYB transcription factor are created upstream of the TAL1 oncogene by heterozygous somatic mutations. This MYB interaction attracts CBP, resulting in the formation of a super-enhancer that promotes cell transformation and leukemogenic expression by driving the overexpression of TAL1.^388^ Similar to this, p300 has been associated with a substantial reprogramming of super-enhancers in HCC, which results in the upregulation of critical oncogenes such MYC, MYCN, and CCND1, which promotes cancer cell proliferation both in vitro and in vivo.^389^

Due to their important role in tumorigenesis, chromatin remodeling complexes (CRCs), especially the SWI/SNF family, are crucial in the DNA damage response (DDR). More than 20% of cancers have mutations in the SWI/SNF complex genes, highlighting the significance of these genes in the genesis of cancer.^390^ Gene mutations can be caused by environmental factors such as UV light and gamma radiation that damage DNA. Prompt damage detection, repair signaling, repair factor mobilization, and cellular fate direction towards apoptosis or senescence are done by the DDR machinery. The historical background of DDR deficits in carcinogenesis is well established, having begun with the finding of chromosomal abnormalities in genes and continuing through the revelation that inadequate telomere maintenance catalyzes genomic instability and the detection of important tumor-suppressive functions of DDR components.^391–393^ The SWI/SNF complexes affect DNA repair pathways by increasing nucleosome mobility through ATPase activity, which facilitates DDR. There are several roles that SWI/SNF subunits play in DDR. Some directly recruit DDR proteins, whereas others alter the chromatin architecture at DNA damage sites.^394–396^ The PBAF and cBAF complexes are associated with DNA repair processes such as homologous recombination (HR) and non-homologous end joining.^337,397^ To be more precise, DNA lesions are attracted by SMARCA4 and the cBAF-exclusive ARID1A, which help in double-strand break resolution and repair.^398^ Poly-ADP ribose polymerase 1 (PARP1) and SMARCA4 have been shown to collaborate at damage sites, promoting chromatin remodeling to lower nucleosome density and assisting in the repair process.^396^ SMARCA4 and ARID1A deficiencies are linked to mitotic abnormalities and erratic chromosomal segregation, suggesting that these proteins also play roles in DNA decatenation and telomere cohesion.^399^ DDR also involves PBRM1, a component of PBAF. It is involved in centromeric cohesion maintenance, which is essential for genomic integrity, and transcriptional suppression at double-strand breaks to speed DNA lesion repair.^400^ CHD1L plays a key role in the regulation of checkpoint control following DNA damage. It facilitates the movement of nucleosomes, which is promoted by PARP1, and also regulates checkpoint activities.^401^ A lack of CHD1L impairs the accessibility of chromatin and the recruitment of repair factors, resulting in increased sensitivity to PARP.^402^ The multifaceted involvement of cancer highlights their pivotal role in maintaining genomic integrity and preventing cancer. Understanding these complex interactions and mechanisms not only elucidates cellular physiology but also provides insights into potential therapeutic targets for cancer treatment.

Topologically Associating Domains (TADs) borders breaking down in the oncogenic landscape represent a fundamental abnormality. These disruptions are often caused by structural variations or damaged CCCTC-binding factor (CTCF) interaction due to changes in DNA methylation.^403^ The proto-oncogene TAL1 is activated by microdeletions that obliterate TAD boundaries in T-ALL.^403^ Broken genomic insulation in gliomas and gastrointestinal stromal tumors interferes with CTCF anchoring at loop structures. Like with PDGFRA and FGF4, this results in oncogene activation and ectopic enhancer-promoter crosstalk.^404,405^ An explanation for the observed correlation between increased CCNE1 expression in gastric cancer primary tumors and CCNE1 rearrangement in response to changed TAD borders and interactions has been found.^406^ Because the promoter-enhancer looping dynamics are dysregulated, this rearrangement promotes oncogenicity. An example of such a dynamic is the interaction between the MYC gene and the lncRNA PVT1 promoter. The PVT1 promoter blocks the promoter-enhancer looping of MYC, which in healthy cells decreases MYC expression competitively. On the other hand, malignant transformation often silences the PVT1 promoter by structural or epigenetic changes, re-establishing MYC’s enhancer-gene interaction and quickening tumorigenesis.^407^ The loss of a single CTCF allele, which has been linked to oncogenic drivers in cancers including breast and endometrial, has supported CTCF’s role as a tumor suppressor through numerous genetic aberrations.^408–410^ Prostate, ovarian, and breast cancers^411^ frequently have hemizygous deletions of CTCF, while kidney and endometrial cancers with an allelic loss of CTCF are linked to lower patient survival.^412,413^ Since hypermethylation of CpG islands is associated with a decrease in CTCF binding, this tumor-suppressing mechanism may entail the regulation of DNA methylation patterns. On top of that, PD-L1 upregulation is associated with CTCF deficiency, which allows cancer cells to evade immune monitoring.^414^

Histone H3 and H4 acetylation patterns have become characteristic indicators of cancer cells, and dysregulation in the histone modification landscape is becoming more and more associated with metastasis. Histone acetylation states in cancer are impacted by metabolic reprogramming, which modifies the absolute amounts of acetyl-CoA and the ratio of acetyl-CoA to coenzyme A. Because of its role in acetyl-CoA gene rating through the ligation of acetate and CoA, ACSS2 can cause HIF-2 to become acetylated, which inhibits EMT under hypoxic conditions in HCC.^415^ Overexpression of ACSS2, which promotes acetylation of H3K27 in the ATG5 promoter region, is used to achieve reduced breast cancer cell proliferation, migration, and invasion. This, in turn, ensures the maintenance of autophagic flow.^416^ ACOT12, also referred as cytoplasmic acetyl-CoA hydrolase, is a predominant liver enzyme that selectively hydrolyzes the thioester bond of acetyl-CoA, gene rating acetate and CoA.^417^ Reduced levels of ACOT12 in HCC lead to increased levels of acetyl-CoA, which in turn promotes the acetylation of H3K9 and H3K56. This acetylation process facilitates the EMT mediated by TWIST2.^418,419^

Acetyl groups are transferred from acetyl-CoA to lysine residues by histone acetyltransferases (HATs). This process helps neutralize the positive charge on histones, thereby loosening the interaction between histones and DNA. As a result, genes become more accessible to transcription factors, facilitating gene expression.^420^ GCN5, the initial identified HAT, controls a diverse array of biological processes including cellular proliferation, gene expression, and metabolism. It has also been demonstrated to play a role in the growth and metastasis of cancer cells.^421^ GCN5 is recruited to the Runx2 promoter to sustain H3K27ac levels, which leads to the upregulation of Runx2 and promotes lung metastasis in osteosarcoma.^422^ The biological function of HDACs in cancer is well-established. HDACs have the ability to both promote and inhibit tumor metastasis. Specific types, mainly through facilitating the downregulation of E-cadherin, have been linked to the proliferation and potential for metastasis of a variety of cancers. These include HDAC1, HDAC2, HDAC4, HDAC5, and HDAC6.^423,424^ Simultaneously, HDAC8 has been identified as a new TGF-β pathway regulator. It works by transcriptionally suppressing SIRT7 via specific chromatin remodeling. Lung cancer metastasis is accelerated by this HDAC8 activity, which functions as a cofactor to the SMAD3/4 complex and triggers the activation of TGF-β signaling.^425^ Histone H3K27 can be maintained in a deacetylated state by recruiting HDAC1 to the promoter of the DUSP2 gene. This results in DUSP2 being silenced and elevated MMP2 levels, promoting metastasis of nasopharyngeal metastesis.^426^ In colorectal cancer, HDAC11 suppresses metastasis by downregulating MMP3 expression. This occurs through the reduction of histone H3K9 acetylation at the MMP promoter.^427^ On the progression and metastasis of breast cancer, HDAC11 may have divergent effects. The survival and proliferation of tumors within the lymph nodes can be enhanced by increased HDAC11 expression, whereas a migratory phenotype is promoted by reduced HDAC11 expression, which significantly increases migration from the lymph nodes to distant organs.^428^

#### Dysregulation in the landscape of histone modifications in CTCs immune escape

CTCs employ various strategies to evade immune surveillance by cytotoxic T cells. These mechanisms include suppressing T-cell activation, altering the expression of MHC-I at both transcriptional and posttranscriptional levels, reducing the levels of TAAs, and overexpressing IC molecules such as PD-L1 and GAL-9.^429^ Downregulation of MHC-I molecules hinders CD8 + T-cell activation against TAAs presented by CTCs. The WNT/β-catenin signaling pathway regulates STT3, which glycosylates and stabilizes PD-L1, leading to increased PD-L1 levels on CTCs and facilitating evasion from cytotoxic T-cell immune surveillance.^430^

In hypoxic conditions within the TME, PD-L1 and VEGF expression is elevated in CTCs. VEGF promotes the expression of TIM-3, the T-cell inhibitory receptor. Interaction between overexpressed PD-L1 and Gal-9 on CTCs with their corresponding receptors (e.g., PD-1 and TIM-3) on T cells suppresses T-cell proliferation, reduces cytokine production, induces T-cell exhaustion, and ultimately enables CTC evasion from cytotoxic T-cell activity.^429,431^ Mature dendritic cells (DCs) are pivotal in initiating T-cell-mediated immune responses by presenting TAAs and expressing costimulatory molecules.^432^ However, CTCs employ diverse mechanisms to inhibit DC-mediated antitumor responses, including the release of TGF-β. Specifically, CTCs hinder the recruitment of CD103+ DCs to tumors,^433^ impair their maturation, promote differentiation into immunosuppressive regulatory DCs,^434^ and induce the development of PD-1+ DCs that deactivate CD8 + T cells.^435^ Through its negative regulation of DCs, macrophages, and T cells expressing their receptors, CD200, an increased immune checkpoint in CLL, BRCA, NSCLC, and COAD, develops immunological tolerance.^436^ The interaction between TAMs and CTCs promotes CTC survival and the establishment of an immunosuppressive TME.^437^ Numerous cells, including fibroblasts, endothelial cells, and immune cells, make up the CTC niche within the TME. These cells are enriched with factors like periostin (OSF-2, an osteoblast-specific factor), TGF-β, and colony-stimulating factor 1.^438^ These factors drive the polarization of macrophages towards an immunosuppressive M2 or TAM phenotype.^439^ TAM-secreted molecules like WNT, TGF-β, and VEGF promote cancer stemness, create an immunosuppressive TME, facilitate EMT, and support cancer metastasis.^440^ Additionally, TAMs enhance PD-L1 expression on CTCs and PD-1 on T cells, thus dampening T-cell-mediated cytotoxicity.^441^

MDSCs play a pivotal role in the TME by secreting cytokines and chemokines that foster an immunosuppressive niche, thereby impairing the efficacy of immunotherapy.^442^ Granulocyte-macrophage colony-stimulating factors are released by CTCs via the mTOR signaling pathway, promoting MDSC infiltration into tumors.^443^ Within the TME, MDSCs release IL-6 and nitric oxide, which epigenetically upregulate CTCs markers such as EpCAM, while also facilitating the activation of Tregs through TGF-β release.^444,445^ By interacting with the immunosuppressive TME, Tregs, an immunosuppressive T-cell fraction, promote CTC immune evasion, suppressing the effects of cancer immunotherapy. CTC-derived TGF-β facilitates Treg infiltration into tumors, correlating with poorer survival rates.^446^ Additionally, CTCs upregulate CCL1 expression through epigenetic mechanisms that decrease H3K27me3 levels at the CCL1 promoter, enhancing Treg recruitment to the TME.^447^ Moreover, CTCs evade T-cell-induced apoptosis by inducing the differentiation of uncommitted CD4 + T cells into Tregs.^448^ Specifically, Tregs in the hypoxic TME release VEGF, promoting CTCs stemness, angiogenesis, and EMT.^449,450^ Natural killer (NK) cells express receptors like NKG2D, FASL, and TRAIL, which selectively target and eliminate MHC-I-negative CTCs.^451^ NK cell-mediated cytotoxicity has been observed in MHC-I-negative colon and ovarian CTCs expressing NKG2DL and ligands for activating receptors NKp30 and NKp44.^452^ However, CTCs from some ovarian and renal carcinoma patients upregulate MHC-I molecules, reducing susceptibility to NK cell-mediated lysis.^444,445^ Interestingly, latent competent cancer cells expressing SOX2/SOX9 induce dormant CTCs (or latency-competent cancer cells) that downregulate NKG2DL through a unique mechanism involving WNT inhibitor DKK1, thereby evading NK cell-mediated immunity.^446^ Epigenetic modifications, such as H3K27 acetylation, are enforced by CBP and p300 in the regulatory regions of genes crucial to Treg cell and MDSC survival and function. Through upregulating these genes, CBP/p300 promotes the growth of tumors by inhibiting lymphocyte activation, proliferation, and immunity mediated by cytotoxic T-cells.^453^ Additionally, it has become clear that the sirtuin family of NAD+-dependent deacetylases, which includes mitochondrial SIRT3, SIRT4, and SIRT5, is essential for regulating epigenetic changes like as acetylation, demalonylation, and desuccinylation.^454^ It has been demonstrated that SIRT4 loss enhances breast cancer stem cells’ capacity for self-renewal, which is essential for nutritional catabolism.^455^ Increased cancer cell proliferation has been associated with desuccinylation and consequently decreased activity of succinate dehydrogenase (SDH), which has been linked to high levels of SIRT5 activity. In contrast, SDH hyper-succinylation and reactivation result from silencing SIRT5, which inhibits the growth of cancer cells.^456^ Interferon serves as a potent cytokine with antitumor properties, inhibiting the expansion of CTCs and suppressing their tumor-initiating capabilities.^448^ IFN-stimulated genes also play a role in overcoming chemoresistance in CTCs.^448^ However, CTCs can develop resistance mechanisms against IFN’s antitumor effects, thereby promoting their survival and inducing the expression of CTC markers while evading immune surveillance through IFN signaling pathways.^447,449^ The dual role of IFNs in cancer therapy may vary depending on the duration and concentration of IFN exposure.^450,451^ Suboptimal type-I IFN signaling triggered by immunogenic cell death (ICD) does not consistently result in effective anticancer immunity. Instead, it can paradoxically promote tumor progression by increasing the population CTCs equipped with enhanced immune evasion capabilities.^452^ Under specific conditions, elevated levels of IFN-γ have been demonstrated to trigger apoptosis in NSCLC through activation of the JAK1/STAT1/caspase pathway. Conversely, low concentrations of IFN-γ can enhance the properties of CTCs through the ICAM1/PI3K/AKT/NOTCH1 pathway, potentially contributing to tumor progression.^457^ Overall, the interplay between various immune cells and CTCs fosters an immunosuppressive TME that facilitates immune evasion, thereby leading to adaptive resistance against cancer immunotherapy.

## Clinical research progress targeting CTCs and therapeutic advances

Current methodologies for eradicating metastasis mirror those utilized for primary tumors: focusing on proliferation and tumorigenesis rather than directly addressing the metastatic cascade.^458,459^ Surgical intervention or systemic treatments for primary tumors may not always eradicate the source of metastasis if dissemination have already happened.^460–463^ The majority of anticancer agents undergo initial evaluation in metastatic contexts before being repurposed for adjuvant therapy to deter metastatic spread, albeit with only moderate success.^464^ The scarcity of agents specifically targeting metastasis is being confronted by numerous preclinical investigations and considerations for future clinical trial frameworks.^1,459^ Theoretically, therapies aimed at CTCs at different points in the metastatic process could halt the progression of metastatic cancers as CTCs are the cause of metastatic cancers and may come from different subpopulations within tumors.

### CTCs in clinical trials

As a preventive approach against metastasis in preclinical models, addressing hypoxia-induced cluster release with vascular normalization-inducing drugs (e.g., ephrin B2 Fc chimaera protein, modulating VEGFR signaling) has been proposed.^465^ The PLK1 inhibitor BI 2536 also impedes CTC intravasation,^165^ suggesting its clinical utility in mitigating metastatic dissemination.^466^ Targeting integrins, cadherins, cell-surface glycoproteins,^467^ invadopodia (e.g., through N-WASP inhibition),^468,469^ or employing antibodies against CD36, P-selectin, αIIbβ3, and α6β1 integrins could prevent intravasation and extravasation. HPSE, which facilitates ICAM1-mediated cell adherence in CTC clusters, is the target of one newly developed drugs class.^470^ Additionally, urokinase and Na^+^/K^+^-ATPase inhibitors like digoxin exhibit promise in dissociating CTC clusters, leading to metastasis suppression in animal models.^5^ Digoxin is now under studied in a phase I trial to determine its potential to disrupt CTC clusters in patients with advanced or metastatic breast cancer (*NCT03928210*). Through cell-cell dissociation, heterotypic clustering may also be disrupted. Blocking key platelet receptors on CTCs, like glycoprotein Ib–IX–V and glycoprotein VI, reduces the potential for metastasis by interfering with platelet–cancer cell interactions.^108^ Similarly, targeting VCAM1 on CTC–neutrophil clusters retards proliferation and metastatic efficiency.^111,471–473^ Alternatively, utilizing CTCs’ VCAM1-mediated affinity for neutrophils for immune-based targeting could imitate the lethal activity of NK cells by energizing neutrophils with nanoscale liposomes that carry TRAIL and E-selectin.^474^ Metabolic vulnerabilities could be exploited by either increasing oxidative stress or inhibiting pyruvate metabolism.^475–477^ Immune checkpoint inhibitors have the ability to identify CTCs for T cell destruction,^478^ and when combined with immune checkpoint inhibitors, dual targeting of HER2 or EpCAM results in improved cancer cell killing as compared to monotherapy.^479,480^ Additionally, by employing mechanically disrupted CTCs as nanolysates, CTCs can be used as a source to produce cancer vaccines. Harnessing the homing capacity of CTCs to TME could be therapeutically advantageous by identifying homing signals and delivering therapeutic payloads.^126,481^ Systemically administered CTCs engineered to express the prodrug-converting enzyme cytosine deaminase-uracil phosphoribosyl transferase can convert non-toxic 5′-fluorocytosine into the cytotoxic compound 5′-fluoruridine monophosphate. This conversion causes the CTCs to attach to the neoplastic tissues, where they kill the surrounding cancer cells. Lastly, given the rhythmic nature of CTC release into the bloodstream,^126,481^ making the most of the current therapeutic opportunities by timing chronotherapy-based designs to coincide with CTC production peaks could optimize efficacy. Although these methods show promise in future research on CTCs, there is a lack of specific tools for dealing with metastatic cells, and the metastatic process is complicated, so testing their effectiveness in clinical settings will necessitate innovative and bold trial designs.

The results of clinical trials have demonstrated that CTCs are found in peripheral blood in all major carcinoma types, and their significance for prognosis in colorectal, breast, prostate, and small- and non-small-cell lung malignancies has been validated.^482,483^ When a patient is first diagnosed with metastatic breast cancer, higher CTC counts before starting treatment are predictive indicators of shorter disease-free and total survival times.^484–486^ For patients with colorectal^487^ and prostate^488,489^ cancer, a negative relationship between pretreatment CTC counts and clinical prognosis has also been reported. Significantly, numerous studies have illustrated that fluctuations in CTC counts following treatment administration offer more robust prognostic insights compared to baseline CTC levels. The persistence of CTCs post-therapy correlates with a poorer prognosis.^490–492^ In patients receiving therapy, evaluation of CTC cluster abundance significantly improves the prognostic value in addition to single-CTC counts.^493^ However, since most of the studies used antigen-dependent CTC methods, enumeration carries the risk of producing false-negative results in this situation.

CTC counts can be detected 7–9 weeks before the disease’s clinical manifestation. This suggests that CTC analysis in patients may help predict the likelihood of minimal residual disease and relapse in later stages,^163^ of the illness^163,494^ and serve as a tool for early cancer diagnosis. In NSCLC, CTCs taken following surgery showed a strong mutational concordance with metastatic tumors found 10 months later (91%).^156^ Although CTCs are useful for risk assessment, there has been limited success in using CTCs for therapeutic patient stratification. This includes monitoring treatment response over time and identifying the onset of therapy resistance in many clinical trials.^491,495,496^ The METABREAST STIC disease trial highlighted scenarios where CTC count could provide useful advice for therapeutic decisions, even though the interventional SWOG-S0500 trial did not show an advantage of CTC count-guided intervention compared to physician’s choice upon disease development.^495^ The benefit of therapy selection based on CTC molecular features has been explored in a number of interventional studies.^497,498^ In two proof-of-principle studies, trastuzumab–emtansine or lapatinib (HER2-targeted treatments) were used to target HER2-positive CTCs in HER2-negative metastatic breast cancer.^498,499^ Although one trial (DETECT III) still awaits completion, the studies have only found a little benefit thus far.^499^ Results from patients receiving endocrine therapy for metastatic prostate cancer are predicted by the expression of AR-V7 in CTCs (PROPHECY trial).^500,501^ A phase II trial was then started to explore how the microtubule inhibitor cabazitaxel affected patients with AR-V7-positive CTCs and metastatic castration-resistant prostate cancer. However, the European Society for Medical Oncology guidelines do not support AR-V7 testing in this context due to the recent negative outcome of that trial, as it offers no advantage over current decision algorithms.^502–504^

In conclusion, CTCs are now included in both the seventh edition of the AJCC Cancer Staging Manual and the fifth version of the WHO Classification of Tumours: Breast Tumours. The designation “cM0 (i+)“ indicates the absence of overt metastasis but the presence of tumor cells in the blood, bone marrow, or lymph nodes. Important cancer societies have yet to incorporate CTCs into their clinical practice recommendations, including the European Society for Medical Oncology and the American Society for Clinical Oncology. Arguably, CTCs’ actual potential lies in their ability to represent highly metastatic tumor subclones and in their abundance as modern biomarkers for molecular and functional studies. CTCs, as living cells, can be cultured outside of the body and analyzed for drugs response, which could offer significant insight to inform treatment choices in a timely manner.^175,505,506^

Despite these advancements, significant enhancements to such workflows are necessary for their transition to clinical application. Innovative, prospective, randomized interventional trials are required to determine whether the use of CTCs as diagnostic aids offers clear benefits over standard-of-care (SOC) methods for specific cancer types. Future validation efforts should give priority to the predicted strengths of CTCs, including their ability to detect little residual illness, express clinically actionable targets for therapy selection, and provide longitudinal follow-up.

### Diagnostic and prognostic value of methylation patterns of CTCs

The investigation of DNA methylation patterns in CTCs spans multiple cancer types, offering insights into their potential as biomarkers for diagnosis, prognosis, and therapeutic targeting.^97,126^ Madhavan et al. highlighted the importance of methylation patterns in circulating cell-free DNA as markers of tumor progression and response to treatment in their investigation of CTC DNA’s potential as a prognostic diagnostic in metastatic breast cancer.^507^ Cabel et al. investigated the role of CTCs and circulating tumor DNA in prostate cancer, focusing on DNA methylation markers as potential tools for assessing disease progression and therapeutic response.^508^ Widschwendter et al. demonstrated that the methylation status of circulating tumor DNA can be used as a non-invasive diagnostic marker for ovarian cancer, enabling early detection and therapeutic response monitoring.^509^ Powrózek et al. examined SHOX2 gene methylation in CTCs of NSCLC patients and discovered that SHOX2 methylation could function as a non-invasive biomarker for NSCLC prognosis and diagnosis. This suggests the possible utility of methylation analysis in CTCs for clinical purposes.^510^ The potential of circulating tumor DNA to identify EGFR mutations in lung cancer patients was examined by Hulbert et al.^511^ In identifying individuals who may benefit from treatment with tyrosine kinase inhibitors, the study demonstrated the utility of circulating tumor DNA as a feasible alternative to invasive tissue biopsies. Furthermore, the research shed light on the expansion of genetic and epigenetic profiling using circulating tumor material, such as exploring DNA methylation patterns. This highlights the growing importance of utilizing non-invasive biomarkers for precision medicine applications in oncology.^511^ Mazor et al. demonstrated how the methylation patterns of circulating tumor DNA in lung cancer patients can serve as indicators of tumor burden and heterogeneity.^512^ Their study underlines the potential of DNA methylation profiling of circulating tumor DNA, encompassing CTC-derived DNA, in providing crucial prognostic and diagnostic insights.^512^ Aberrant DNA methylation profiles have been detected in CTCs from cancer patients, and these patterns have shown promise as diagnostic or prognostic biomarker. For instance, Wong et al.’s study, which examined the DNA methylation patterns of CTCs from lung cancer patients, found a correlation between patient survival and the methylation levels of specific genes.^513,514^ Specifically, high methylation of HOXA9 and LMX1A genes was associated with poor overall survival, while high methylation levels of the IGFBP3 gene were associated with better overall survival.^515,516^ Another study by Wu et al. investigated the DNA methylation patterns of CTCs from patients with lung adenocarcinoma and identified differentially methylated genes that were associated with metastasis.^5,111,126^ They identified that the methylation levels of GABRB2, CLDN3, and SFRP1 were conspicuously different between CTCs from patients with and without metastasis.^517^ In addition to their potential as diagnostic or prognostic biomarkers, DNA methylation patterns in CTCs may also have therapeutic implications. For instance, a study by Chen et al. suggested that treatment with a DNA-demethylating agent called decitabine reduced the metastasis of CTCs from lung cancer patients by reversing the aberrant DNA methylation patterns in these cells.^332,518^ The DNA methylation profile of CTCs has demonstrated a significant potential for lung cancer diagnosis, prognosis, and treatment (Table 4).^495,519–525^

**Table 4:** Application of DNA methylation of CTCs in lung cancer

Application	Genes	DNA methylation level	References
Early detection	PCDHGB7	Hypermethylation	^519^
Prognosis	RASSF1A and APC	methylation	^520^
Predicting treatment response	MGMT	methylation	^521^
Resistance to targeted therapy	PTEN	Hypermethylation	^522^
Tumor heterogeneity	\	methylation	^523^
Tracking disease progression	HOXA9	methylation	^524^
Minimal residual disease detection	RASSF1A and APC	methylation	^525^
Personalized treatment	\	methylation	^495^

### DNA methylation detection in CTCs

The principal methodology utilized in liquid biopsy is the identification of DNA methylation within the circulatory system. Circulating tumor DNA has better sensitivity and specificity for cancer screening than traditional tumor markers, especially in the early stages of the disease. The sensitivity of Vimentin gene methylation in serum for the diagnosis of CRC was found by Atsushi Shirahata et al. to be significantly higher (57.1% vs. 14.3%) for diagnosing CRC in situ (Stage 0) than for the tumor marker CEA (32.6% vs. 33.1%).^432^ Similar to this, the FDA has approved SEPT9, a circulating tumor DNA test, as an effective early non-invasive screening method for colorectal cancer.^526,527^ SEPT9, also referred to as MSF, was originally discovered by Osaka et al.^530^ They observed that MSF acts as a proto-oncogene, promoting leukemia upon fusion with the MLL gene.^528^ Further research has shown that SEPT9 generates 18 transcriptional products, each contributing uniquely to the development and progression of cancer.^529^ Specifically, the SEPT9 gene, particularly the SEPT9_v2 variant, functions as a tumor suppressor in colorectal cancer.^530^ Hypermethylation of the SEPT9_v2 promoter region, leading to decreased SEPT9 gene expression, is a defining characteristic of colorectal cancer (CRC) and is closely associated with the progression from adenoma to atypical hyperplasia to CRC. In their 2021 study, Guoxiang Cai et al. developed a classifier named “ColonAiQ”, which incorporates six circulating tumor DNA markers (SEPT9, SEPT9 region 2, BCAT1, IKZF1, BCAN, VAV3).^531^ Their findings showed that “ColonAiQ” was able to achieve a greater detection rate in both early and advanced CRC than fecal immunochemical tests, CEA, and SEPT9.^531^ Additionally, the “ColonAiQ” classifier predicted early postoperative recurrence and poor prognosis of CRC, with patients exhibiting higher ColonAiQ risk scores more likely to experience early postoperative recurrence.^531^ Using whole-genome bisulfite sequencing technology, Liu et al. conducted a prospective clinical trial to examine tissue and circulating tumor DNA methylation in breast cancer.^423^ They found methylation patterns that differed between cancer patients and those with benign tumors, and they developed a diagnostic model. The detection of breast cancer was significantly improved by this combined diagnostic models. One benefit of the study is that it uses whole-genome bisulfite sequencing to identify differential methylation sites in detail. This method measures the average degree of DNA methylation at each CpG site in the target genome. However, this method does not precisely pinpoint methylation sites. A variety of methods have been used in investigations to identify site-specific DNA methylation, including digital PCR, pyrosequencing, bisulfite sequencing, and methylation-specific PCR.^532–534^ The study’s examination of methylation differences in tissues and circulating tumor DNA from cancer and benign lesion patients enables the exclusion of non-cancer-related methylation sites in circulating tumor DNA. Differential diagnosis on patients with benign masses improves the detection of tumor-specific sites in contrast to many other studies that employ healthy controls. Additionally, combining imaging examinations (e.g., ultrasound, mammography) with circulating tumor DNA testing improves breast cancer detection sensitivity and specificity, reducing unnecessary invasive procedures. This prospective clinical trial focused on early-stage cancer lesions, offering insights distinct from studies utilizing advanced cancer tissues, which might not be as effective for early cancer screening. Nevertheless, the study had limitations, such as unmatched patient age, smoking, and other influencing factors between case and control groups, potentially introducing heterogeneity and affecting site detection efficacy.^535,536^ Moreover, the study’s small sample size (10 tissue pairs) diminished locus selection efficiency, likely influenced by the high cost of whole-genome sequencing. Therefore, developing cost-effective sequencing technologies is crucial for early cancer screening. Colonoscopy, ultrasonography, mammography, and low-dose chest CT have become widely used and have greatly improved early screening for lung cancer, breast cancer, and colorectal cancer, as well as early diagnosis rates and patient prognoses.^537–539^ However, effective screening tools for pancreatic cancer (PC) remain lacking. Nine studies on blood-based DNA methylation biomarkers for early PC diagnosis, all published in the last decade, have been retrieved.^533,540–544^ Notably, three studies identified ADAMTS1 methylation as a liquid biopsy marker for PC, achieving high diagnostic efficacy (sensitivity >80%, specificity >85%).^540,545,546^ Keiko Shinjo et al. also demonstrated the diagnostic capability of a five-DNA molecule panel, including ADAMTS2, for PC, with sensitivity and specificity of 68 and 86%, respectively, when combined with KRAS mutation.^542^ The ADAMTS family, comprising 19 members, plays crucial roles in arthritis, cardiovascular diseases, and cancer,^547^ particularly in regulating ECM structure and function. Given the abundant stroma in PC, certain ADAMTS family subtypes may serve as effective biomarkers for PC diagnosis, warranting further investigation.^548^ The circulating tumor DNA methylation model is also pivotal in prognosis. Mastoraki et al. discovered that non-small cell lung cancer patients with methylation in the KMT2C promoter region of circulating tumor DNA experienced poorer overall survival (OS) and disease-free survival (*P* = 0.017 and *P* < 0.001, respectively).^549^ Promising results have been found in numerous studies examining the role of circulating tumor DNA in predicting the prognosis of different cancers, including colorectal, prostate, and ovarian cancer.^549–555^ Establishing a prognostic model based on blood-based DNA methylation facilitates early identification of high-risk groups, enabling timely and effective intervention, while non-high-risk individuals may undergo milder treatment or follow-up, thus minimizing unnecessary invasive treatments and conserving medical resources. Despite the utility of circulating tumor DNA methylation in reflecting patient prognosis across various cancers, challenges remain in developing prognostic methylation markers. The lack of consensus on detection methods and the difficulty in identifying universally accepted prognostic methylation sites contribute to these challenges. In order to determine prognosis and choose adjuvant treatment, DNA gene mutation data derived from tumor tissue or blood is widely used. The clinical application of assessing the methylation status of the MGMT gene promoter in gliomas, which indicates tumor response to temozolomide chemotherapy and patient prognosis, has been successful.^556,557^ Unfortunately, this application is based on tumor tissue-derived MGMT gene promoter methylation status, with no mature clinical studies on circulating tumor DNA.

### Therapeutic targeting of methylation in CTCs

To evaluate the effectiveness of treatment and track any tumor recurrence, tumor markers and cross-sectional exams are widely used in the postoperative follow-up of cancer patients. After surgery, a decrease in serum tumor markers indicates effective treatment, whereas an increase indicates a possibility of metastasis or recurrence.^558,559^ Tumor marker detection has been shown to predict tumor recurrence up to six months ahead of cross-sectional imaging^560^; however, higher tumor markers are correlated with tumor burden and may not be visible in the early stages of relapse.^561^ Other factors can also elevate tumor marker levels; for example, CA19-9, a reliable marker for monitoring postoperative PAAD recurrence, can be elevated due to pancreatic inflammation, obstructive jaundice, and persistent diabetes.^558^ Approximately 8–10% of the population are Lewis-negative, and over 70% of Lewis-negative PAAD patients exhibit low CA19-9 expression, rendering this marker ineffective for prognosis in these patients who typically have worse outcomes.^562^ Because it is noninvasive, highly reproducible, and sensitive, the detection of circulating tumor DNA methylation has become a crucial method for dynamically monitoring tumor response after treatment.^563^ In order to highlight the importance of circulating tumor DNA in cancer surveillance, Michail Ignatiadis et al. introduced the term “circulating tumor DNA relapse”.^564^ Nakayama et al. found that P16INK4a methylation is a sensitive marker of colorectal cancer recurrence, highlighting the crucial role circulating tumor DNA plays in the postoperative follow-up of CRC patients.^565^ In their study of 21 CRC patients, 13 exhibited elevated P16INK4a methylation pre-surgery, with all patients showing decreased methylation levels within 2 weeks post-surgery, except for two with residual metastases or subsequent relapse. In these two patients, there was noticeably higher P16INK4a methylation at relapse; this was not the case in the individuals without tumor recurrence.^565^ Jin et al. found that cfDNA follow-up could detect colon cancer recurrence early, with circulating tumor DNA reappearing in 70% of patients (14 cases) before recurrence, approximately eight months earlier than imaging suggested.^566^ Therefore, early tumor relapse detection is made possible by dynamic monitoring of circulating tumor DNA methylation, which aids in clinical decision-making. The tumor information provided by circulating tumor DNA aids in guiding subsequent targeted therapy selection. Tumor heterogeneity partially explains the poor response to antitumor therapy, with new clonal subtypes forming during tumor progression, a significant factor in therapeutic inefficacy.^567^ Circulating tumor DNA detection provides great reproducibility when compared to standard tissue biopsy, which decreases the effects of tumor tissue heterogeneity and allows for timely treatment plan adjustments and dynamic monitoring of therapy response. However, compared to traditional tissue exams, circulating tumor DNA extraction and sequencing involves greater technical demands and expenses.

Current research primarily focuses on mutation information within circulating tumor DNA. Detailed gene mutation analysis can elucidate the cancer molecular landscape, potentially leading to more suitable treatment options. However, studies on using circulating tumor DNA methylation for therapeutic target selection are scarce. This disparity might result from the theory that tumor responses rather than tumor causes are the reason for variations in the circulating tumor DNA methylation state in cancer patients. More research is needed in this area. DNA methylation in tissues is critical for tumor suppressor gene inactivation and tumorigenesis. Cancers including pancreatic, breast, and bladder cancer have demonstrated benefit from targeted therapy of DNA methylation, notably when DNA methyltransferase inhibitors are used.^568–570^

### Therapeutic targeting of histone modification enzyme in CTCs

Beyond broad-spectrum modifiers, drugs designed to target particular mutations within enzymes that modify the epigenome are becoming part of the landscape of epigenetic therapies. One such drug is tazemetostat, a selective inhibitor that specifically targets the EZH2 mutation. EZH2, the PRC2 complex’s catalytic component, regulates transcriptional repression via H3K27 trimethylation. Overexpression of EZH2 is associated with poor prognosis and increased malignancy in various cancers,^571^ prompting its exploration as a therapeutic target. Based on phase 2 trial results that demonstrated a 69% objective response rate (ORR) in patients with EZH2 mutations, compared to 35% in those with wild-type EZH2, Tazemetostat was approved by the FDA.^572^ Dual inhibitors that target both EZH1 and EZH2 have been found to be more effective than selective EZH2 inhibition in reducing cellular H3K27me3 levels and increasing anticancer effects in mouse models of hematologic malignancies.^573^ In a phase 2 trial, the dual inhibitor valemetostat demonstrated promise in treating adult T-cell leukemia/lymphoma, with a 48% ORR.^574^ Currently, a phase 1 trial for metastatic urothelial cancer is exploring the possible synergistic effects of valemetostat with ipilimumab. In a similar vein, DOT1L, the sole H3K79 methyltransferase, has been studied as a possible therapeutic target in tumors containing MLL gene rearrangements, particularly in cases of acute leukemia.^575^ Even though the outcomes of early clinical trials of DOT1L inhibitors were inconsistent, pinometostat may make juvenile AML cells more sensitive to the multikinase inhibitor sorafenib, opening the door to novel therapeutic options.^576^ These developments are poised to reshape the therapeutic landscape for pediatric AML and highlight the evolving precision in targeting specific epigenetic mutations for cancer therapy.

The advancement of cancer has been linked to abnormal LSD1 amplification and activity.^577^ LSD1’s role in transcriptional repression involves removing methylation from H3K4me1/2, a marker of gene activation.^578^ Preclinical evidence of differentiation and growth attenuation has prompted the evaluation of LSD1 inhibitors, including pulrodemstat (CC-90011), iadademstat, seclidemstat, and GSK2879552, in a number of clinical trials. Results from early phase studies, notably those employing pulrodemstat for solid tumors and non-Hodgkin lymphoma, show significant anti-neoplastic effects, particularly in neuroendocrine tumors.^579^ Several non-histone proteins, including as DNMT1, are impacted by LSD1 in addition to histones.^580^ LSD1-mediated demethylation of DNMT1 is critical for its stabilization and the maintenance of global DNA methylation patterns.^581^ LSD1 inhibitors have the potential to be used in combination therapy for hematological malignancies. A clinical trial, with the ClinicalTrials.gov code NCT04734990, is presently exploring the use of seclidemstat in combination with azacytidine for the treatment of chronic myelomonocytic leukemia. Preclinical studies have also demonstrated that by raising tumor immunogenicity and T-cell infiltration, LSD1 inhibition can improve the effectiveness of immune checkpoint blockade. Clinical trials exploring combination therapies have been started to optimize the effects of immunotherapy, particularly in tumor forms with previously limited responses, including lung cancer.^582,583^ These developments underscore the expanding potential of LSD1 inhibitors in both standalone and combination treatments across a spectrum of cancers.

HDACs have zinc-enriched active sites, which HDAC inhibitors (HDACi) bind to in order to impede their function and maintain a hyperacetylated state of chromatin that promotes a transcriptionally active configuration.^584^ In order to treat cutaneous T-cell lymphoma, the FDA approved vorinostat, one of the first-generation HDACi, in 2006. This approval was based on clinical trials demonstrating ORRs of ~30%.^585^ Similar to DNMT inhibitors, HDACi have shown synergistic effects in preclinical studies when combined with other anticancer agents, leading to the strategic design of combination clinical trials. HDAC inhibitors not only enhance the expression of PD-L1, potentially priming tumors for immunotherapy, but also reduce populations of Tregs, which can bolster immune responses against tumors.^586^ Vorinostat’s capacity to make hormone-resistant ER-positive breast tumors more susceptible to apoptosis has been validated in preclinical studies, indicating that it may be used in combination with antiestrogen drugs to improve therapeutic results in hormone therapy.^587^ In 2014, Belinostat, a second-generation HDAC inhibitor, obtained expedited FDA approval for the treatment of peripheral T-cell lymphoma based on the results of a single-arm trial with 120 patients.^588^ To address toxicity concerns related to earlier generations of HDACi, selectivity against specific members of the HDAC family has been improved through advancements in HDAC inhibition. A benzamide derivative called entinostat has shown promise as a strong and specific inhibitor of class I and IV HDACs. Low-dose azacitidine plus entinostat has been explored in clinical studies for patients with advanced breast cancer and recurrent metastatic NSCLC, particularly in those who have had extensive prior treatment.^589,590^ These studies reflect ongoing efforts to optimize the efficacy and safety profiles of HDAC inhibitors in cancer therapy.

Proteins called bromodomain and extraterminal (BET) domains are important chromatin dynamics regulators and have become promising targets in cancer research. The BRD2, BRD3, BRD4, and BRDT proteins belong to the BET protein family and use their bromodomains to identify acetylated lysine residues on histones. This recognition initiates chromatin remodeling and gene expression by recruiting additional transcriptional effectors.^591^ A key factor in identifying the oncogenic functions of BET proteins has been the emergence of small-molecule BET inhibitors like JQ1. These inhibitors disrupt the binding of BET proteins to acetylated histones, thereby modulating the expression of key oncogenes implicated in cancer progression.^592,593^ Despite their initial promise in preclinical models, the clinical translation of BET inhibitors has been limited by pharmacokinetic challenges, including short half-lives and poor oral bioavailability. Tyrosine kinase inhibitors like lapatinib can be resistant to BET inhibitors like JQ1 and I-BET151 in TNBC. In order to prolong the therapeutic response, they achieve this by suppressing the production of certain kinases which drive resistance mechanisms.^594,595^ Furthermore, homologous recombination, a crucial DNA damage repair pathway, is disrupted by BET inhibitors by the transcription of proteins involved. This disruption has significant implications for cancer therapy, particularly in combination approaches. When PARP inhibitors and BET inhibitors are used together, tumors that are proficient in homologous recombination may become more sensitive to the PARP inhibitors and may eventually acquire resistance to them.^596,597^ Clinical investigations have been prompted by the synergistic benefits between PARP and BET inhibition observed in preclinical studies, particularly in breast and ovarian cancers. In order to provide novel avenues for targeted cancer therapy, these studies aim to validate these results in patient populations.^598,599^ This strategic combination approach highlights the potential of BET inhibitors to enhance the efficacy of existing therapies and to address resistance mechanisms in cancer treatment.

## Conclusion and perspective

Cancers remain the highest number of cancer-related deaths globally. CTCs are tumor cells that have separated from the primary tumor and entered the lymphatic or circulatory system. This allows the tumor cells to spread throughout the body and result in the formation of new tumors. According to studies, patients with lung cancer can have CTCs in their blood, and the number of CTCs in a patient’s blood is correlated with the disease’s progression and chances of metastasis. CTCs have been found in the blood of lung cancer patients even before a tumor has been identified by conventional methods, providing evidence that they may be a valuable tool for the early identification and monitoring of lung cancer.

DNA methylation is the addition of methyl groups to the cytosine residues of DNA, and it is necessary for regulation the expression of certain genes. Numerous cancer types, including lung cancer, have been related to abnormal DNA methylation patterns at the onset and progression of the disease. It has been discovered that DNA methylation has a role in both immune surveillance and metastasis in the setting of CTCs.^600^ The immune system’s method of identifying and getting rid of cancer cells is called immune surveillance. However, by DNA methylation, CTCs can suppress the expression of immune-related genes, enabling them to elude the immune system and persist in the bloodstream. For example, some CTCs have been found to have DNA hypermethylation of genes that are involved in antigen processing and presentation, which may help them escape recognition and elimination by the immune system. Furthermore, DNA methylation has also been involved in the metastasis of CTCs. The EMT process, for example, is essential for cancer cell invasion and metastasis, and it has been linked to DNA hypermethylation of E-cadherin, a gene involved in cell adhesion and migration. Additionally, it has been found that the metastatic potential of CTCs^273,601^ is influenced by hypomethylation of genes related to DNA repair and cell cycle regulation.^273,601^

There are several limitations that must be addressed before CTC analysis can be widely employed in the clinic, despite the fact that it has showed promise in the diagnosis and treatment of lung cancer. One of the major limitations is the low sensitivity of CTC detection methods. The number of CTCs in the bloodstream is usually very low, and current methods for CTC detection and isolation may miss a significant proportion of these cells. This may limit the clinical value of CTC analysis and lead to false negative results. The diversity of CTCs presents another difficulty. In contrast to the primary tumor or other CTCs, CTCs are a diverse population of cells that may exhibit various phenotypic and genetic characteristics. This heterogeneity can make it difficult to identify and isolate CTCs that are representative of the entire tumor and to develop targeted therapies.^602,603^ Furthermore, CTCs are often found in a dormant state, meaning they are not actively dividing or producing detectable levels of tumor markers. This can make it challenging to monitor the response of the tumor to therapy using CTC analysis alone. Finally, there are technical challenges correlated with CTC analysis, such as the need for specialized equipment and expertise, which may limit the availability and accessibility of this technology to all patients. Addressing these limitations may require the development of new technologies and methods for the detection and analysis of CTCs as well as an improved comprehension of CTC biology and role in lung cancer.^604,605^

This review has encapsulated the obstacles surrounding the genesis of CTCs in the context of cancers, as well as the effect of epigenetics modifications on CTCs pertaining to EMT, immune surveillance, cluster formation, and colonization. The epigenetic modifications resulting from DNA methylation in CTCs may serve as a key to unlock the underlying mechanisms of metastasis in lung cancer, and holds significant promise in the areas of lung cancer diagnosis, prognosis, and treatment.
